# Structure of human immunoproteasome with a reversible and noncompetitive inhibitor that selectively inhibits activated lymphocytes

**DOI:** 10.1038/s41467-017-01760-5

**Published:** 2017-11-22

**Authors:** Ruda de Luna Almeida Santos, Lin Bai, Pradeep K. Singh, Naoka Murakami, Hao Fan, Wenhu Zhan, Yingrong Zhu, Xiuju Jiang, Kaiming Zhang, Jean Pierre Assker, Carl F. Nathan, Huilin Li, Jamil Azzi, Gang Lin

**Affiliations:** 1 Cryo-EM Structural Biology Laboratory, Van Andel Research Institute, Grand Rapids, MI 49503 USA; 2 Department of Biochemistry and Milstein Chemistry Core Facility, Weill Cornell Medicine, New York, NY 10065 USA; 3 Transplantation Research Center, Renal Division, Brigham and Women’s Hospital, Harvard Medical School, Boston, MA 02115 USA; 4 Department of Microbiology & Immunology, Weill Cornell Medicine, New York, NY 10065 USA; 5 National Center for Macromolecular Imaging and Marrs McLean Department of Biochemistry and Molecular Biology, Baylor College of Medicine, Houston, TX 77030 USA

## Abstract

Proteasome inhibitors benefit patients with multiple myeloma and B cell-dependent autoimmune disorders but exert toxicity from inhibition of proteasomes in other cells. Toxicity should be minimized by reversible inhibition of the immunoproteasome β5i subunit while sparing the constitutive β5c subunit. Here we report β5i-selective inhibition by asparagine-ethylenediamine (AsnEDA)-based compounds and present the high-resolution cryo-EM structural analysis of the human immunoproteasome. Despite inhibiting noncompetitively, an AsnEDA inhibitor binds the active site. Hydrophobic interactions are accompanied by hydrogen bonding with β5i and β6 subunits. The inhibitors are far more cytotoxic for myeloma and lymphoma cell lines than for hepatocarcinoma or non-activated lymphocytes. They block human B-cell proliferation and promote apoptotic cell death selectively in antibody-secreting B cells, and to a lesser extent in activated human T cells. Reversible, β5i-selective inhibitors may be useful for treatment of diseases involving activated or neoplastic B cells or activated T cells.

## Abstract (web summary)

Immunoproteasome selective inhibitors might lead to less toxic drugs for treatment of multiple myeloma and graft rejection. Here, the authors develop immunoproteasome specific asparagine-ethylenediamine (AsnEDA)-based inhibitory compounds, demonstrate their efficacy and present the AsnEDA bound immunoproteasome cryo-EM structure.

## Introduction

Degradation of most cytosolic proteins is a highly regulated, ATP-dependent cellular activity executed by the ubiquitin-proteasome system (UPS)^1^. The UPS plays essential roles in diverse cellular activities, including cell cycle control, signal transduction, protein homeostasis and immune surveillance. The degradation machinery of the UPS, the 26 s proteasome, is composed of a hydrolytic barrel-like 20 s core and regulators, such as 19 s or 11 s, on either or both ends of the 20 s. The 20 s core that is constitutively expressed in most cells (c-20S) is a stack of 4 rings of 14 α and 14 β subunits organized in a α_1–7_β_1–7_β_1–7_α_1–7_ fashion, where 2 copies of each caspase-like β1, trypsin-like β2 and chymotrypsin-like β5 active subunit are located in the inner β rings^2^. The chymotrypsin-like β5 active subunit of the 20 s has been clinically validated as a target for the treatment of multiple myeloma and certain lymphomas. The Food and Drug Administration-approved drugs bortezomib and carfilzomib represent two classes of covalent proteasome inhibitors: reversible peptide boronates and irreversible peptide epoxyketones, respectively^3^. Several other classes of proteasome inhibitors have been identified and optimized, such as β-lactones and peptide sulfonyl fluorides^4^. However, their reactive warheads pose a challenge for developing a drug candidate. Several noncompetitive proteasome inhibitors have recently been reported^5–7^.

We have been developing isoform-selective, non-covalent inhibitors for various proteasomes, such as the *Mycobacterium tuberculosis* proteasome^8–12^ and the human immunoproteasome (i-20 s)^13–15^. I-20S is expressed in cells of the immune system and other cells exposed to cytokines that are elevated during immune responses, where the active subunits β1c, β2c, and β5c in c-20S are replaced by β1i, β2i and β5i, respectively^16–18^. The i-20S serves diverse functions in the immune system, including the provision of oligopeptides for antigen presentation, T cell differentiation and proliferation^19,20^. Antibody-secreting plasma cells are highly sensitive to proteasome inhibition. Bortezomib, which inhibits both c-20s and i-20S, has been used in renal transplant recipients to treat antibody-mediated graft rejection^21^. Bortezomib was also reported to be efficacious in patients with refractory systemic lupus erythematosus^22^. However, bortezomib’s substantial mechanism-based toxicity requires use of much reduced doses in the treatment of non-malignant conditions.

To regulate immune responses through proteasome inhibition with less mechanism-based toxicity to immune cells and little or none to other cells, it would be useful to inhibit i-20S selectively, sparing c-20S. Consistent with this notion, and unlike disruption of genes encoding c-20S subunits, disruption of genes encoding β1i, β2i and β5i results in mice that are healthy, fertile and immunocompetent^23^. Indeed, relatively selective inhibition of β5i over β5c with the compound ONX-0914 has been efficacious in several mouse models of autoimmune disease^24^. However, ONX-0914 belongs to the peptide epoxyketone class of inhibitors whose irreversible mechanism involves recruiting the hydroxyl and amino groups of the active site Thr^1N^ into formation of a 1,4-oxazepane adduct with the epoxyketone warhead. Long-term use of an irreversible inhibitor presents a risk of toxicity from the gradual, cumulative inhibition of c-20S and potentially of other targets. Therefore, it would be desirable to develop inhibitors that are highly selective for i-20S as well as reversible^25^. Several recent studies have reported the development of β5i-selective inhibitors^26,27^. Because of the abundance of proteasomes’ substrates, an additional benefit might accrue from a noncompetitive mode of action, so that progressive accumulation of substrate does not lessen the degree of inhibition. Herein we report the serendipitous discovery of a class of non-covalent compounds that noncompetitively and selectively inhibit β5i over β5c.

## Results

### Scaffold morphing from dipeptides to AsnEDAs

We recently reported a class of irreversible inhibitors that selectively inhibit the *Mycobacterium tuberculosis* proteasome over human c-20S^11^. We later found that this class of inhibitors also selectively inhibits i-20S over c-20S^13^, reflecting that the mycobacterial and human i-20S proteasomes share similar folding around the β5i active subunit. We later identified non-covalent N,C-capped dipeptides with bulky hydrophobic aromatic rings at P1 and N,N- dual substituted asparagine side chains that are highly potent and selective for Mtb20S over both human c-20S and i-20S^12,28^. We then developed a class of N,C-capped dipeptidomimetics by incorporating a β-amino acid into the N,C-capped dipeptides, which resulted in markedly potent and selective inhibitors for β5i over β5c^15^. We also developed β5i-selective N,C-capped dipeptides that suppressed T-cell activation and proliferation in vitro and helped prevent rejection of cardiac allografts in mice^14^.

**Fig. 1:**
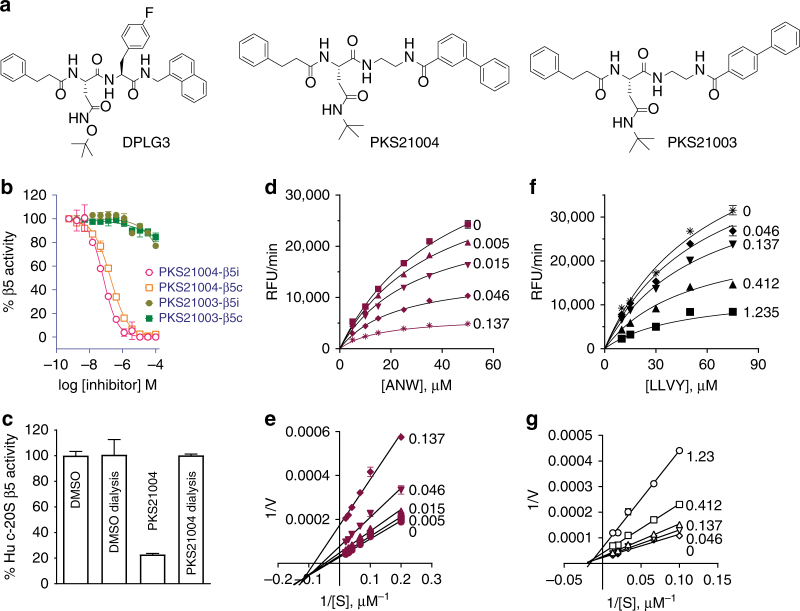
AsnEDAs noncompetitively inhibit β5i and β5c. **a** Dipeptide DPLG3 and AsnEDA PKS21004 and PKS21003. **b** PKS21004 potently and dose-dependently inhibits β5i and β5c, whilst PKS21003 is inactive. **c** PKS21004 reversibly inhibits β5c. **d**–**g** kinetics studies of modality of inhibition for PKS21004 against β5i (**d**, **e**) and β5c (**f**, **g**). **d** Steady state velocities in the presence of PKS21004 at the concentrations indicated next to each curve. Data as in **d** are shown in double reciprocal plots in **e**, and data from **f** in **g**. Values of Ki and α for PKS21004 were determined to be 0.077 µM and 0.28 µM for β5i, and 0.55 µM and 0.65 for β5c, respectively, by fitting the data to an equation for noncompetitive inhibitors. Data are means ± SEM of three independent experiments

To move away from peptides, we systematically replaced the amide bonds in select N,C-capped dipeptides with bioisosteres. Reversing the amide bond at the C-cap and replacing the amino acid with an aromatic carboxylic acid resulted in a chemotype: Asn-ethylenediamine (AsnEDA) (Fig. 1a). The first compound of this class, PKS3080, yielded modest IC50s of 0.37 and 1.22 µM against human β5i and β5c, respectively. Replacement of the 1-naphthoyl with 2-naphthoyl (producing PKS21025) improved potency against β5i by 3-fold and increased selectivity to 26-fold.

Replacement of the *N*-cap 1-naphthoyl with [1,1′-biphenyl]-4-carboxamide (PKS21003) and [1,1′-biphenyl]-3-carboxamide (PKS21004) drastically impacted activity. PKS21003 had no detectable activity against either β5i or β5c (IC50 > 100 µM for both), whereas PKS21004 was a potent inhibitor of both β5i and β5c, with IC50s of 0.058 and 0.326 µM, respectively (Fig. 1b). Inhibition of the β5 subunits was specific, as no inhibition was observed of β1 or β2 activities in either i-20S or c-20S (Supplementary Table 1).

### Modality of inhibition

As expected from their non-covalent chemistry, dialysis of a pre-incubated mixture of c-20S and PKS21004 fully restored β5c activity (Fig. 1c), confirming the reversibility of this class of inhibitors. Kinetic analysis indicated that PKS21004 inhibits β5i and β5c noncompetitively with substrate. With increasing concentrations of PKS21004, both *V*_max_ and *K*_M_ decreased (Fig. 1d–g and Supplementary Fig. 1). This indicates that inhibition of β5i and β5c by PKS21004 is of a mixed noncompetitive type with α_c-20S_ ≈ 0.65 and α_i-20S_ ≈ 0.28^29^, and that PKS21004 binds more tightly to both β5c and β5i with substrate bound than without substrate (Supplementary Fig. 1). Decreasing *V*_max_/*K*_M_ with increasing PKS21004 concentration also suggests that PKS21004 is not an uncompetitive inhibitor of either 20S (Supplementary Fig. 1)^29^. This class thus joins a growing list of noncompetitive proteasome inhibitors^5,30^.

**Fig. 2:**
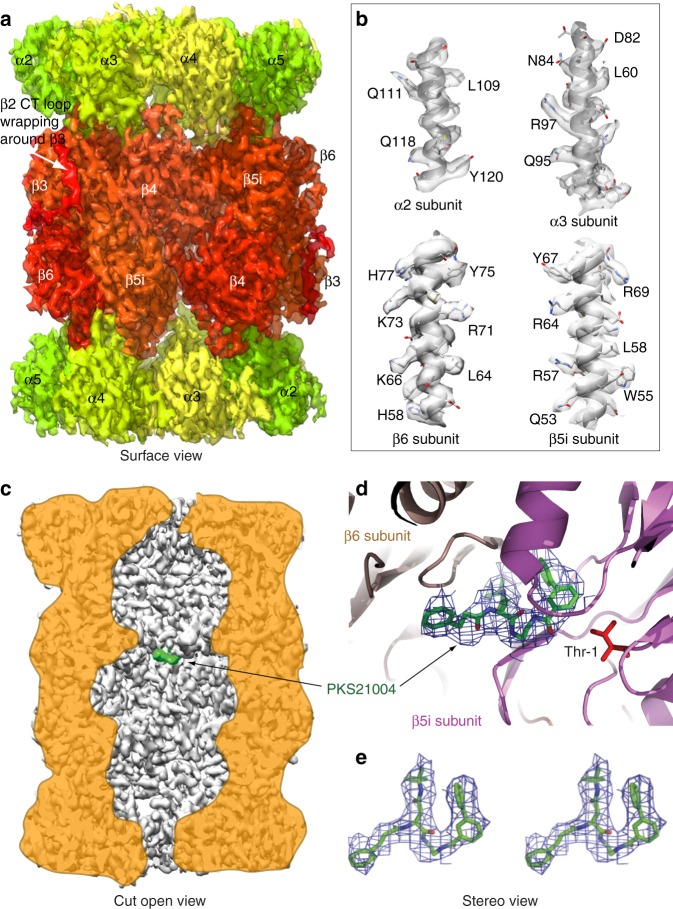
Structure of the human i-20S bound to PKS21004, determined by high-resolution cryo-EM and single-particle analysis. **a** Surface view of the cryo-EM map with each subunit colored differently. **b** Electron densities of four selected α-helices demonstrating the resolution of many side chains of the i-20S complex. **c** A vertical central section of the 3.8-Å cryo-EM density map rendered as surface view. The inhibitor density is highlighted in green. **d** The electron density of the PKS21004 in the β5i active site shown as blue meshes with modeled atomic structure shown as green sticks. The structure of the β5i is shown as cartoon in magenta and β6 in salmon, respectively. The proteolytic residue Thr1 of β5i is in red sticks. **e** A stereo view of the binding pose of PKS21004

### Cryo-EM structure of human i-20S with PKS21004

To identify the binding site of the compound, we used high-resolution cryo-electron microscopy and single-particle analysis to determine the structure of the human immunoproteasome in complex with PKS21004 (See Experimental methods). The final cryo-EM 3D map had a resolution of 3.8 Å in which the secondary structures such as β-strands and α-helices were very well resolved (Fig. 2a, Supplementary Figs. 2–6). Many large side chains were also resolved (Fig. 2b). The large side chain densities, in conjunction with the previously published crystal structures of the human constitutive proteasome (PDB code 5LF1)^25^ and the mouse immunoproteasome (PDB code 3UNH)^31^, enabled us to build an atomic model at this resolution (see Table 1 for model statistics). In the electron density map and the atomic model, the very long carboxyl terminal extension of the human β2i embraces nearly half of the β3 subunit (Fig. 2a); this feature is highly conserved from yeast to humans (Supplementary Fig. 7).

**Table 1:** Cryo-EM data collection and refinement statistics

Human i-20S-PKS21004 (EMD-7010, PDB 6AVO)	
*Data collection*	
EM equipment	JEOL JEM-3200FS
Voltage (kV)	300
Detector	Gatan K2 Summit
Pixel Size (Å)	1.2
Electron Dose (e^−^ Å^−2^)	60
Underfocus range (µm)	1.0–3.0
*Reconstruction*	
Software	RELION 2.0
Number of particles used	75,017
Resolution (Å)	3.8
Map-sharpening B factor (Å^2^)	124.3
*Model composition*	
Peptide chains	28
Protein residues	6115
Ligands	2
*R.m.s deviation*	
Bond length	0.009
Bond angle	1.146
*Ramachandran plot*	
Preferred (%)	90.70
Allowed (%)	9.22
Outlier (%)	0.08
*Validation*	
MolProbity score	2.52
Good rotamer (%)	98.02
Clashscore, all atoms	32.47

We were able to unambiguously assign the density in the substrate-binding cleft near the active site of β5i to PKS21004 (Fig. 2c–e). No additional densities were observed in the active site of any other β-subunits or in any of the α− subunits. This result is consistent with the conclusion that PKS21004 specifically inhibits the activity of the β5 subunits. PKS21004 binds to human i-20S between β5i and β6 at a position similar to where the covalent inhibitor dihydroeponemycin binds in the β1c, β2c, and β5c of the human c-20S (PDB code 5LF1)^25^.

**Fig. 3:**
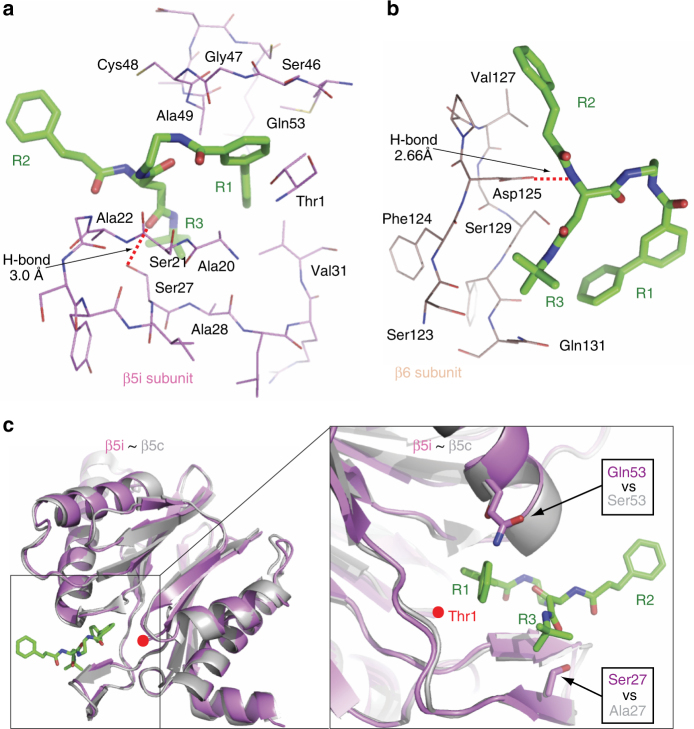
The interaction of PKS21004 with the human i-20S. **a** Detailed hydrophobic interactions between the R1 and R3 groups of PKS21004 with the substrate pocket of the human β5i subunit. A hydrogen bond between Ser27 and the oxygen atom of the PKS21004 R3 group (3.0 Å) is shown as a dashed red line. **b** Interactions of the R2 and R3 groups of PKS21004 with the neighboring β6 subunit. The strong H-bond between Asp125 and the nitrogen atom of the R2 of PKS21004 (2.66 Å) is shown by a dashed red line. **c** Representation of the PKS21004 (green sticks) in the β5i active site (purple cartoon). The structure of β5 (gray cartoon, PDB 5LF1) is superimposed for comparison. The right panel is an enlarged and rotated view of the area in the square box of the left panel. Red dots denote the active residue Thr1 of β5 and β5i. The two residues in human β5i that make contact with the inhibitor and are different from the human β5c are shown in stick and marked by two black arrows

PKS21004 is largely hydrophobic and interacted with β5i and β6 primarily via hydrophobic contacts (Fig. 3a–b, Supplementary Fig. 8A, B). Specifically, the R1 group extended deeply into the S1 and S1 sub pocket (S1SP), forming extensive hydrophobic contacts with Ala20, Val31, Ser46, Gly47, Ala49, and Gln53 of β5i, and Tyr130 of β6; the R2 group, being outside of the active site, interacted with Ser21, Ala22, and Cys48 of β5i, and Val127 of β6; the R3 group was next to the β6 subunit, forming interactions with Ala28 of β5i, and Ser123, Phe124, Ser129, and Gln131 of β6. The inhibitor also formed two hydrogen bonds, one between the R3 group and Ser27 of β5i, and the other between R3 and Asp125 of β6. The overall structures of the catalytic β1i, β2i, and β5i are very similar to their constitutive counterparts, except for several minor differences in the loop regions (Supplementary Fig. 9A). However, among the human β5i residues interacting with PKS21004, many are different from their corresponding residues in the human β5c (Supplementary Figs. 8, 9B). Notably, Ala27 and Ser53 in β5c are replaced by Ser27 and Gln53 in β5i, thus disrupting two important interactions with PKS21004 in the β5i (Fig. 3c, Supplementary Fig. 8A, C). The side chain of Met45 is swung outward as compared to the apo human β5c or β5c in complex with CFZ to accommodate the bulky R1 group in β5i. (Supplementary Fig. 10). These differences may explain why PKS21004 selectively inhibits i-20S over c-20S.

The seeming discrepancy between kinetic and structural studies indicates that PKS21004 belongs to a class of active site-directed noncompetitive inhibitors, along with certain inhibitors of other enzymes, especially kinases^32^. However, to our knowledge, this is the example of an active site-directed noncompetitive inhibitor of proteasomes.

### Optimization of AsnEDAs

The foregoing features of these AsnEDA compounds encouraged a further round of SAR studies (Table 2) in which we varied the carboxylic acid at the ethylenediamine, the *N*-cap at the Asn, and the side chain of Asn, of PKS21004. All compounds listed in Table 1 were synthesized as illustrated in Scheme 1–9 in Supplementary Methods. The structures of all final compounds were confirmed by nuclear magnetic resonance (NMR) and high-resolution mass spectroscopy (HRMS). IC_50_s of all compounds against β5i and β5c (Table 2) and against β1i, β2i, β1c, and β2c (Supplementary Table 1) were determined following reported methods^8^. All compounds were specific for the β5 subunit; no inhibition of β1i, β1c, β2i, or β2c was observed. Comparison of the ethylenediamine with a methyl-ethylenediamine (PKS21018) and a 1,3-propyldiamine (PKS21019) indicated that the ethylenediamine gave the greatest potency for β5i and selectivity over β5 c. We then modified the [1,1′-biphenyl]-3-carboxamide of PKS21004 with the following substituents: 4′-F (PKS21026), 4′-cyano (PKS21028), 4-F (PKS21196), 4,3′-diF (PKS21195) and 4,2′-diF (PKS21187). The 4′-substitutions decreased potency, while 4- and 4,3′- substitutions did not. Best, 4,2′-diF- (PKS21187) improved potency against β5i to IC50 0.015 µM and improved selectivity to ~20-fold over inhibition of β5c. Consistent with these results, the binding pocket in the cryo-EM structure is large enough to accommodate the new fluoride in 4- and 4,3′- positions. Notably, two crucial residues around these sites, Ser46 and Gln53 in β5i, are changed to Ala46 and Ser53 in β5c, which may account for the improved potency of PKS21187 against β5i over β5c (Supplementary Fig. 8A–D).

**Table 2:** SAR studies and IC50s of compounds against human β5i and β5c

All IC50s are means of at least three independent experiments, except that IC50s greater than 10 µM are means of two independent experiments

Next, we investigated a *N*-Boc-Asp-^*t*^Bu substitution on PKS21277, an intermediate in the synthesis of PKS21187. Compared with PKS21187, PKS21277 has a smaller R2 group, which may abolish the interactions with Val127 in β6 and Cys48 in β5i that is changed to Gly48 in β5c (Supplementary Fig. 8A–C). Consistently, PKS21277 was modestly potent against β5i, with 36-fold selectivity over β5c (Table 2, Supplementary Fig. 8D). Replacing the Asp-^*t*^Bu with Gly (PKS21276), Ala (PKS21280), or Asn (PKS21281) abolished the inhibitory activities against both β5i and β5c, suggesting that Asp-^*t*^Bu is critical for optimal binding to β5 (Supplementary Fig. 8). We then replaced phenylpropionate with tosyl on the *N*-cap of the Asn of PKS21187, yielding PKS21221. Based on the cryo-EM structure, the tosyl group may form tighter interactions with Ser27 and Cys48 in the human β5i (Supplementary Fig. 8). IC50s were determined to be 4 nM against β5i and 110 nM against β5c, representing 30-fold selectivity. Again, replacing the Asp-^*t*^Bu of PKS21221 with Gly (PKS21289), Ala (PKS21290), or Asn (PKS21288) eliminated inhibitory activity against both β5i and β5c. We then replaced the tosyl of PKS21221 with acetyl which yielded PKS21294. PKS21294 showed much reduced potency in comparing to PKS21221 by 150-fold against β5i, by 120-fold against β5c. Replacing the tosyl of PKS21221 with cyclopropanesulfonyl, yielded PKS21293 that showed 3.7-fold reduction in potency against β5i in comparing to PKS21221. However, its selectivity against β5i over β5c was improved from 27.5-fold to 64-fold.

To corroborate that the noncompetitive modality of inhibition was shared among this class of compounds, we tested PKS21221 against β5i and confirmed the noncompetitive mechanism; *α* was determined to be 0.26 (Supplementary Fig. 11), in agreement with that of PKS21004.

### PKS21221 selectively induces cell death in MM cells

**Fig. 4:**
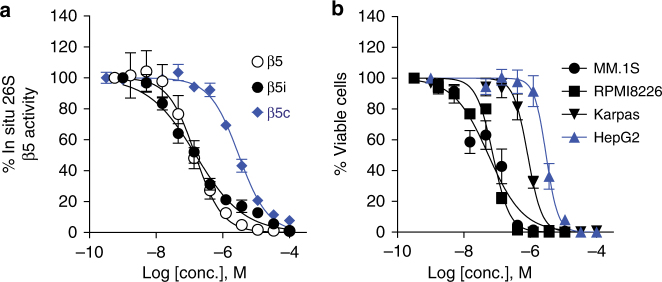
Intracellular proteasome inhibition by PKS21221 and its cytotoxicity against transformed cell lines. **a** Karpas 1106P lymphoma cells (white and black symbols) or HepG2 cells (blue symbols) were treated with PKS21221 for 2 h at the concentrations indicated prior to incubation with substrate (Ac-ANW)_2_R110 for β5i (Karpas cells) or suc-LLVY-luciferin for β5 (Karpas cells and HepG2 cells). IC50s were 0.154 and 0.149 µM for the two substrates in Karpas cells. **b** Cytotoxicity of PKS21221 against multiple myeloma cell lines MM.1S (solid circle) and RPMI 8226 (solid square), Karpas (solid triangle) and HepG2 (blue triangle). Cell viability was determined by CellTiter-glo. Values of IC50 of intracellular proteasome inhibition and EC50 of cytotoxicity are listed in the Table 3. Data are means ± SEM of three to four independent experiments

To determine if this class of inhibitors is cell penetrable, we treated the B-cell lymphoma line Karpas 1106P^28^ (expressing a high proportion of i-20S over c-20S) with PKS21221 before incubation with either (Ac-ANW)_2_-R110 (a specific substrate of β5i) or suc-LLVY-luciferin (a substrate of β5) (Fig. 4a). IC50 values with both substrates were almost identical (172 and 118 nM) and indicated the cell-penetrating ability of these compounds. Similarly, PKS21221 inhibited β5c activity in HepG2 hepatoma cells with an IC50 of 1833 nM (Table 3). No β5i activity was detected in HepG2 cells using (Ac-ANW)_2_-R110 as a substrate. PKS21221 itself does not inhibit luciferase activity at 100 µM when tested over the range 0.002–100 µM.

**Table 3:** Inhibition of intracellular proteasomal activities and cytotoxicity of PKS21221

IC50^a^ (nM)			EC50^a^ (nM)			
Karpas β5i	Karpas β5_(i+c)_	HepG2 β5c	MM.1S	RPMI 8226	Karpas	HepG2
172 ± 33	118 ± 33	1833 ± 138	78 ± 30	66 ± 6	740 ± 145	2320 ± 630

β5i activity was assayed with (Ac-ANW)_2_-R110

β5_(i+c)_ and β5c activity were assayed with suc-LLVY-luciferin

β5_(i+c)_ defines the total chymotryptic activity inside the Karpas cells

^a^Data are means ± SEM of three to four independent experiments

Multiple myeloma cells are hypersensitive to inhibition of chymotryptic activity of their proteasomes and the pan-proteasome inhibitor bortezomib has clinical benefit in patients with multiple myeloma^33,34^. We incubated MM.1S, RPMI 8226, Karpas and HepG2 cells with PKS21221 at different concentrations, and determined cell viability after 72 h (Fig. 4b). PKS21221 showed potent cytotoxicity against the myeloma cell lines MM.1S and RPMI 8226, but was ~10-fold and ~30-fold less cytotoxic against the Karpas lymphoma cell line and the HepG2 hepatocarcinoma cell line cells, respectively (Table 3).

### PKS21221 is selectively cytotoxic to ASC’s

**Fig. 5:**
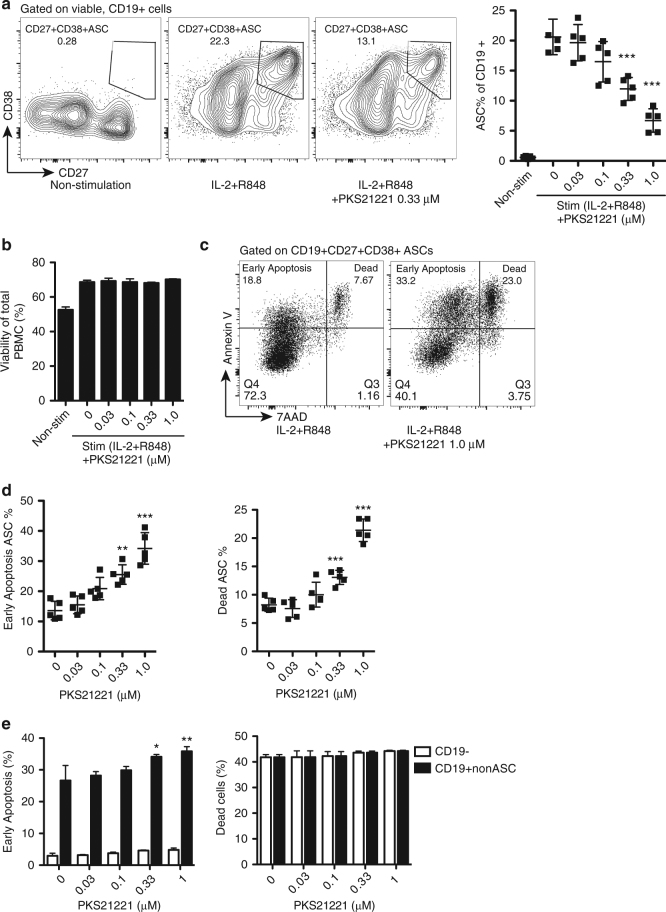
PKS21221 induced apoptosis and cell death in differentiated antibody-secreting cells (ASCs) and CD19^+^ B cells. PBMCs were cultured with or without IL-2/R848 for 5 days, followed by 12-h incubation with PKS21221 at different concentrations. **a** Representative flow cytometry plots of cells treated with IL-2 and R848 in the presence and absence of PKS21221. IL-2 and R848 differentiated B cells into CD27^+^CD38^+^ antibody-secreting cells (ASCs). PKS21221 reduced the percentage of ASCs. **b** Viability of total PBMCs using 7-aminoactinomycin D (7-AAD). 12-h treatment with PKS21221 did not affect overall viability of PBMCs. **c** A representative plot of apoptosis and viability assay using Annexin V and 7-AAD. Annexin V^+^7-AAD^−^ cells were referred as “Early apoptosis” population, and Annexin V^+^7-AAD^+^ cells were referred as “Dead” population. **d** Percentage of early apoptotic ASCs (left) and dead ASCs (right), after 12-h incubation with PKS21221. PKS21221 treatment induced apoptotic cell death in a dose-dependent manner. **e** Early apoptotic (left) and dead (right) populations in CD19^−^ non-B cells and CD19^+^non-ASCs. Experiments were repeated on PBMCs from 5 donors in 5 separate experiments, each data points in **b** and **e**, were the mean + SEM of three technical replicates. *p < 0.05, **p < 0.01, ***p < 0.001, by *t*-test compared with non-treated group

The immunoproteasome plays important roles in T cell differentiation and proliferation and dendritic cell activation and maturation^14,20,24^. However, its role in B cell differentiation is not well understood. The pan-proteasome inhibitor bortezomib depleted plasma cells in mice with lupus-like disease^35^. To investigate whether an immunoproteasome-selective inhibitor impacts the survival of B cells, especially differentiated antibody-secreting cells (ASCs), we differentiated B cells in human peripheral blood mononuclear cells (PBMCs) into CD19^+^CD38^+^CD27^+^ ASCs in the presence of IL-2 and toll-like receptor (TLR)7/8 agonist R848 for 5 days^36,37^. Differentiated ASCs were then exposed to PKS21221 at indicated concentrations and cell survival was determined with Annexin V and 7-aminoactinomycin D (7-AAD) at three-time points (12-h, 24-h, and 48-h after treatment). The percentage of viable ASC decreased with PKS21221 treatment in a dose- and time-dependent manner (Fig. 5a, Supplementary Fig. 12b, c), while the overall viability of PBMCs was not affected (Fig. 5b, Supplementary Fig. 12D). This was explained by enhanced apoptosis (Fig. 5c, d, left) and cell death (Fig. 5c, d, right) of the ASCs. Apoptosis of CD19^+^ non-ASCs was slightly enhanced but no increase was observed in apoptosis of CD19^−^ cells (Fig. 5e, left). Similarly, PKS21221 treatment did not affect viability of CD19^−^ cells or CD19^+^ non-ASCs (Fig. 5e, right). These results suggest that PKS21221 specifically affected the viability of ASCs and to a lesser extent, non-antibody-secreting CD19^+^ B cells. In addition, the cytotoxicity of PKS21221 to different cell populations in PBMC was examined and compared to the cytotoxicity of bortezomib. PBMCs were incubated with PKS21221 or bortezomib, and the viability was assessed in T cells (CD3^+^), B cells (CD19^+^), monocytes (CD14^+^) and dendritic cells (CD3^−^CD19^−^CD14^−^CD16^−^CD11c^+^HLA-DR^+^), respectively (Supplementary Fig. 13b–e). We compared 10 nM bortezomib to 1 μM PKS21221 as these concentrations gave comparable reductions in ASCs in the differentiation culture (Supplementary Fig. 12B). Bortezomib was more cytotoxic at 10 nM than PKS2122 at 1 µM to resting CD3^+^ T cells and CD19^+^ B cells in (Supplementary Fig. 13b, c), suggesting that PKS21221 was more selectively cytotoxic than bortezomib for activated B cells and ASCs.

### PKS21221 inhibits proliferation of T cells

**Fig. 6:**
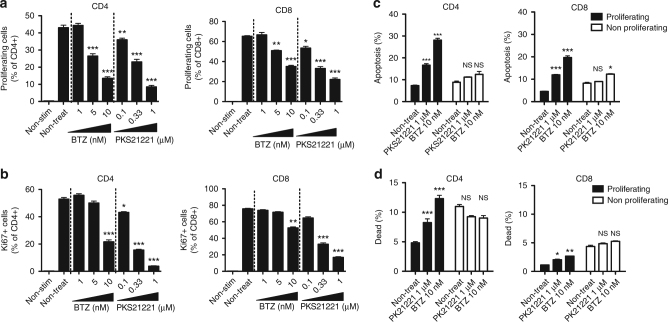
Effect of PKS21221 and bortezomib (BTZ) on T-cell proliferation. T cells were isolated from human PBMC and stimulated with anti-CD3 (1 µg per ml) and anti-CD28 (1 µg per ml) for 4 days. The gating strategy for flow cytometry is shown in Supplementary Fig. 14. **a** The percentage of proliferating cell % of CD4 (left) and CD8 (right) T cells in the presence of different concentrations of BTZ or PKS21221 is shown (mean ± SEM of triplicates). **b** The percentage of Ki67^+^ cell % of CD4 (left) and CD8 (right) T cells in the presence of different concentrations of BTZ or PKS21221 is shown (mean ± SEM of triplicates). Both agents inhibited the proliferation of T cells in a dose-dependent manner. **c** and **d**: T cells were stimulated with anti-CD3 and anti-CD28 for 4 days, followed by incubation with PKS21221 (1 µM) or BTZ (10 nM) for 24 h. Apoptosis **c** and viability **d** were assessed in proliferating and non-proliferating population of T cells. PKS21221 and BTZ induced apoptosis more significantly in proliferating cells, both in CD4 + and CD8 + T cells. Data are representative results with cells from three different healthy donors, each tested in two different independent experiments. *p < 0.05, **p < 0.01, ***p < 0.001, by *t*-test compared with non-treated group

As we observed selective cytotoxicity of PKS21221 in activated B cells and ASCs compared to non-activated B cells, we asked whether this is the case in proliferating T cells. We activated human T cells to proliferate with anti-CD3 and anti-CD28 in the presence or absence of PKS21221 or bortezomib. Both compounds inhibited proliferation of T cells in a dose-dependent manner (Fig. 6a, b). To examine whether these inhibitors are cytotoxic preferentially to proliferating cells, we triggered T-cell proliferation with anti-CD3/anti-CD28 for 4 days (in the absence of inhibitors), treated the cells with PKS21221 for the next 24 h and determined cell survival with Annexin V and 7-AAD (Fig. 6c, d). Both PK21221 and bortezomib induced apoptosis and cell death to a greater extent in proliferating T cells than in non-proliferating T cells, suggesting that proteasome inhibitors preferentially affect survival of activated, proliferating T cells.

## Discussion

These results introduce a versatile new scaffold of proteasome inhibitors, describe their potential for optimization for selective inhibition of the immunoproteasome over the constitutive proteasome, present the cryo-EM structure of the human immunoproteasome in complex with an inhibitor, and demonstrate the inhibitors’ ability to kill myeloma cells, activated human B cells and ASCs in vitro, and to a lesser extent, proliferating human T cells.

To our knowledge, the structure of the human immunoproteasome has not been previously reported, nor has a structure previously been reported of any immunoproteasome with a kinetically noncompetitive inhibitor. The enhanced interior resolution allowed us to confidently interpret the favorable binding of PKS21004 in β5i over β5c and is guiding structure-activity relationship studies to improve potency and selectivity. How active site-binding AsnEDAs kinetically behave like noncompetitive inhibitors remains to be determined. Active site-directed noncompetitive inhibitors of other enzymes have been reported and mechanistic investigations have suggested several possibilities^32^. A molecular dynamics study of the proteasome suggested that conformational changes are induced upon binding of an inhibitor to one β5 that are propagated to the trans-β5 in the other β ring through β4^38^. That mechanism does not appear to apply here, as the Hill coefficient constant is ~1 for the inhibition of either β5i or β5c by either PKS21004 or PKS21221, respectively. It remains to be investigated if binding of inhibitor to one β5 only affects the hydrolytic activity of the trans-β5, but not its binding of substrate.

A growing body of evidence shows that selective inhibition of the immunoproteasome is beneficial in immunity-related diseases without the mechanism-based toxicity of indiscriminate inhibition of all proteasomes. Some immune cells are highly susceptible to proteasome inhibition. For example, plasmacytoid dendritic cells^39^ and plasma cells^40^ are hypersensitive to bortezomib. However, differential hypersensitivity of immune cells towards selective β5i inhibition is unknown. Here we show that malignant myeloma cells are susceptible to β5i inhibition. Furthermore, we found that differentiated ASCs and proliferating T cells are susceptible to β5i inhibition. This suggests the potential clinical utility of β5i-selective inhibitors in the treatment of diseases with dysregulated B-cell activity, such as antibody-mediated allograft rejection or autoantibody-exacerbated disorders such as lupus and type 1 diabetes. Such effects may be enhanced by inhibition of the proliferation of helper T cells.

To our knowledge, this is a reported example of active site-directed noncompetitive proteasome inhibitors. Unlike competitive inhibitors whose intracellular activity is expected to diminish if substrates accumulate, noncompetitive inhibitors are expected to retain a similar degree of inhibitory activity with substrate accumulation, or even increased activity, given that the binding of AsnEDA compounds to β5c and β5i was enhanced by substrate.

## Methods

### Reagents

The human 20S immunoproteasome core sample was purchased from Boston Biochem. The human i-20s at a concentration of 1.5 mg per ml was incubated with PKS21004 dissolved in DMF with a molar ratio of 1:250 ratio for 1 h at 37 °C, then the mixture was diluted in 50 mM HEPES, pH 7.6, 100 mM NaCl and 1 mM dithiotreitol to a final concentration of 0.1 mg per mL for cryo-EM study. Antibodies used for flow cytometry are described as below (clone, company): CD3 (UCHT1, Biolegend), CD4 (OKT4, Biolegend), CD8 (SK1, Biolegend), CD11c (3.9, Biolegend), CD14 (RMO52, Beckman Coulter), CD16 (3G8, Biolegend), CD19 (HIB19, BD), CD38 (LS198–4–3, Beckman Coulter), CD27 (O323, eBioscience), IgD (IA6–2, eBioscience), HLA-DR (L243, Biolegend), Ki67 (Ki-67, Biolegend).

### Cryo-EM

2.8 µL of sample was applied on a carbon-coated Quantifoil R2/1 grid that was freshly glow-discharged in the presence of amylamine to prevent preferred orientations^41^. Furthermore, the grid was plunged into liquid ethane using a FEI Vitroblot IV at 5 °C and 95% humidity with a blot force setting of 5, blot time of 3 s and a wait time of 20 s. The grids were imaged with a 300 kV JEOL JEM-3200FS microscope at a magnification of ×30,000, calibrated pixel size of 1.2 Å and an underfocus range of 1–3 µm. The images were recorded with a Gatan K2 Summit direct electron detector, where each image is composed of 50 individual frames with an exposure time of 10 s and a total dose of 60 e^−^Å^−2^. A total of 2622 micrographs were collected in three sessions.

### Image processing and 3D reconstruction

Images were motion corrected using MotionCor2^42^, followed by removal of bad micrographs by visual screening for drift and high/low defocus. In addition, micrographs where Thon rings did not extend beyond 10 Å were also excluded. Furthermore, particles were picked using swarm from EMAN2^43^ followed by manual removal of false positives and inclusion of false negatives. CTF calculations were done with GCTF^44^ and particle coordinates were imported to RELION 2.0^45^, where all next steps were performed. An initial group of 505,880 raw particles were extracted using a 256 × 256 pixel box size and 2D classification was performed. Particles belonging to the best classes were selected and regrouped, yielding a total of 478,709 particles. A preliminary 3D classification and refinement was performed using the human constitutive 20S proteasome^41^ as initial model and the resulting map was then used for further processing. Further 3D classification followed using a 20 Å low-pass filtered electron density map where initially C1 symmetry was used to remove bad classes, yielding 378,196 particles, and C2 symmetry for further rounds. Next, 3D refinement was performed and a 4.65 Å map was generated as well as a solvent mask. The process was repeated using a solvent mask for 3D classification and 3D refinement, yielding a 4 Å map from 75,017 particles. In addition, movie refinement and particle polishing were performed using a running average of 15 frames. A final round of 3D refinement using the polished particles and subsequent post processing with correction of the modular transfer function of the detector and negative B factor application resulted in a final map of 3.8 Å where the Fourier shell correlation is 0.143. Local resolution was estimated with RELION 2.0^45^.

### Structural modeling with refinement and validation

As an initial model, the crystal structure of human constitutive proteasome (PDB code 5LF1)^25^ was first docked and fitted into the density map using COOT^46^ and Chimera^47^. The structures of β1, β2 and β5 were then replaced by models of human immunoproteasome subunit β1i, β2i, and β5i respectively, which were generated from the crystal structure of the mouse immunoproteasome (PDB code 3UNH)^31^ using the SWISS-MODEL server^48^. The entire human immunoproteasome atomic model was subsequently adjusted manually and rebuilt in COOT. Rigid body and real space refinements of the resulted atomic model were then performed by Phenix^49^. We also performed the reciprocal space refinement procedure with the application of secondary structure and stereochemical constraints in the program Phenix. Finally, the atomic model was validated using MolProbity^50^. Structural figures were prepared in Chimera^47^ and Pymol (https://www.pymol.org). The final model was cross validated using a method described previously^51^, which indicated that our model was not over-fitted.

### Cell-based proteasome β5 activity assay

Intracellular proteasomal activity and inhibition assay were performed as reported^14^. Karpas 1106P (80,000 cells per well) were plated and incubated with compound at indicated concentrations for 1 h at 37 °C. The activity of the overall β5 activity including β5i and β5c in each was measured in situ after compound removal with Proteasome-Glo assay kit according to manufacturer’s instructions. Luminescence was recorded on a SpectraMax M5 plate reader. Relative percentage of RLU was used to calculate the IC50s.

### Malignant B-cell cytotoxicity assays

We used Karpas 1106P B lymphoma cell line (Cat. No. 06072607, Aldrich)^15,16,52^, HepG2 (HB-8065, ATCC), MM.1S (Cat. No. CRL-2974, ATCC). Cells were cultured at 37 °C in a humidified air/5% CO_2_ atmosphere in medium supplemented with 10% fetal bovine serum, except for the medium for Karpas 1106P cells which contained 20% fetal bovine serum, and 100 units per ml penicillin, 100 μg per ml streptomycin in RPMI 1640 medium. Karpas 1106P was used at 80,000 cells per well, MM.1S at 100,000 cells per well, and HepG2 at 12,000 cells per well. Cells plated in a 96-well plate were treated with compounds at indicated concentrations for 72 h at 37 °C in a tissue culture incubator with 5% CO_2_. Viable cells were counted using Cell-titer Glo assay kit. EC50s were calculated using PRISM (Graphpad).

### Antibody-secreting cell differentiation from human PBMC

Human peripheral blood samples were obtained from five different healthy volunteers (BWH Specimen Bank). Peripheral blood mononuclear cells (PBMCs) were isolated by density gradient centrifugation using Lymphoprep (StemCell Technologies). PBMCs were cultured at 1.5 × 10^6^ cells per mL in RPMI 1640 media (Lonza), supplemented with 10% human serum (GemCell) (10% HS-RPMI), recombinant human IL-2 10 ng per mL (or 100 U per ml, BioLegend) and R848 1 µg per mL (Cayman Chemicals) for 5 days, at 37 °C, 5% CO_2_. PKS21221 in DMSO, or DMSO only for control, was added to the culture media on day 5, and cells were incubated for additional 12, 24, and 48 h. Cells were then stained with surface marker antibodies and analyzed by flow cytometry. The viability and apoptosis were assessed using Annexin V Apoptosis Detection Kit (BD Biosciences).

### Viability assay of human PBMC

Human PBMCs were obtained from 5 different healthy volunteers as above. Isolated PBMCs were incubated in 10% HS-RPMI at 5 × 10^5^ cells per 200 μl per well in 96-well round bottom plate (Corning), for 12, 24, 48 h, in the presence of different concentrations of PKS21221 or Bortezomib (Selleckchem). The viability of different populations of PBMCs, such as T cells (CD3^+^), B cells (CD19^+^CD3^−^CD14^−^), monocytes (CD14^+^), dendritic cells (CD3^−^CD19^−^CD14^−^CD16^−^HLADR^+^CD11c^+^), were assessed by flow cytometry using Fixable Viability dye (Themo Fisher).

### In vitro T cells activation and proliferation

T cells were isolated from human PBMCs using Human T-cell Isolation kit (StemCell Technologies, Cat#17951). The purity of T cells was greater than 90% as confirmed by flow cytometry. Cells were counted and loaded with 5 μM CellTrace Violet (Thermo Fisher) in phosphate buffered saline (PBS) for 10 min at 37 °C, followed by wash in 10% fetal calf serum (FCS)-supplemented RPMI 1640 media. T cells were cultured in 10% HS-RPMI, with anti-CD3 (1 μg per mL, eBioscience) and anti-CD28 (1 μg per mL, eBioscience), in the presence or absence of different concentration of PKS21221 or Bortezomib (Selleckchem). In some experiments, T cells were cultured in 10% HS-RPMI / anti-CD3 / anti-CD28 for 4 days and then treated with PKS21221 or Bortezomib (Selleckchem) for 12, 24, 48 h. The viability and apoptosis were assessed using Annexin V Apoptosis Detection Kit (BD Biosciences). The experiments were performed in cells from three different healthy volunteer donors.

### Flow cytometry

Cells were stained with surface marker antibodies at pre-titrated concentrations, in 2% FCS-containing PBS on ice for 30 min. For intracellular staining of Ki67, surface-stained cells were washed with 2% FCS-PBS, followed by incubation in fixation/permeabilization buffer (eBioscience) on ice for 45 min and intracellular staining in permeabilization buffer (eBioscience) for 30 min at room temperature. Cells were analyzed using FACS CantoII (BD Biosciences) and BD FACS Diva software (BD Bioscience). Data were analyzed by FlowJo software (version 10, Tree Star). Gating strategies for each experiment were provided in the supplementary information.

### Data availability

All the relevant data are available within the paper and its Supplementary Information file or from the corresponding authors upon reasonable request. The cryo-EM 3D map has been deposited in the EMDB database with accession code EMD-7010. The corresponding atomic model has been deposited in the RCSB PDB with accession code 6AVO.

## Electronic supplementary material

Supplementary Information
