# Supplementary material for: Structure of human immunoproteasome with a reversible and noncompetitive inhibitor that selectively inhibits activated lymphocytes
Source: Nat Commun. 2017 Nov 22;8:1692. doi: 10.1038/s41467-017-01760-5 (PMC5700161; doi:10.1038/s41467-017-01760-5)
Supplement: Supplementary file 1 — Supplementary Information [file 41467_2017_1760_MOESM1_ESM.pdf]

Supplementary Information

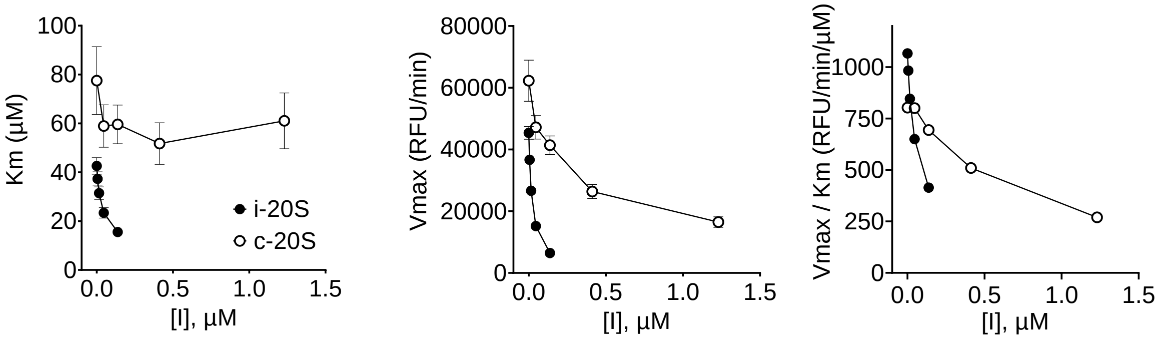

Supplementary Figure 1. Noncompetitive Kinetics of PKS21004 versus β5i and β5c. Kinetic data was shown in Figure 1D-G.

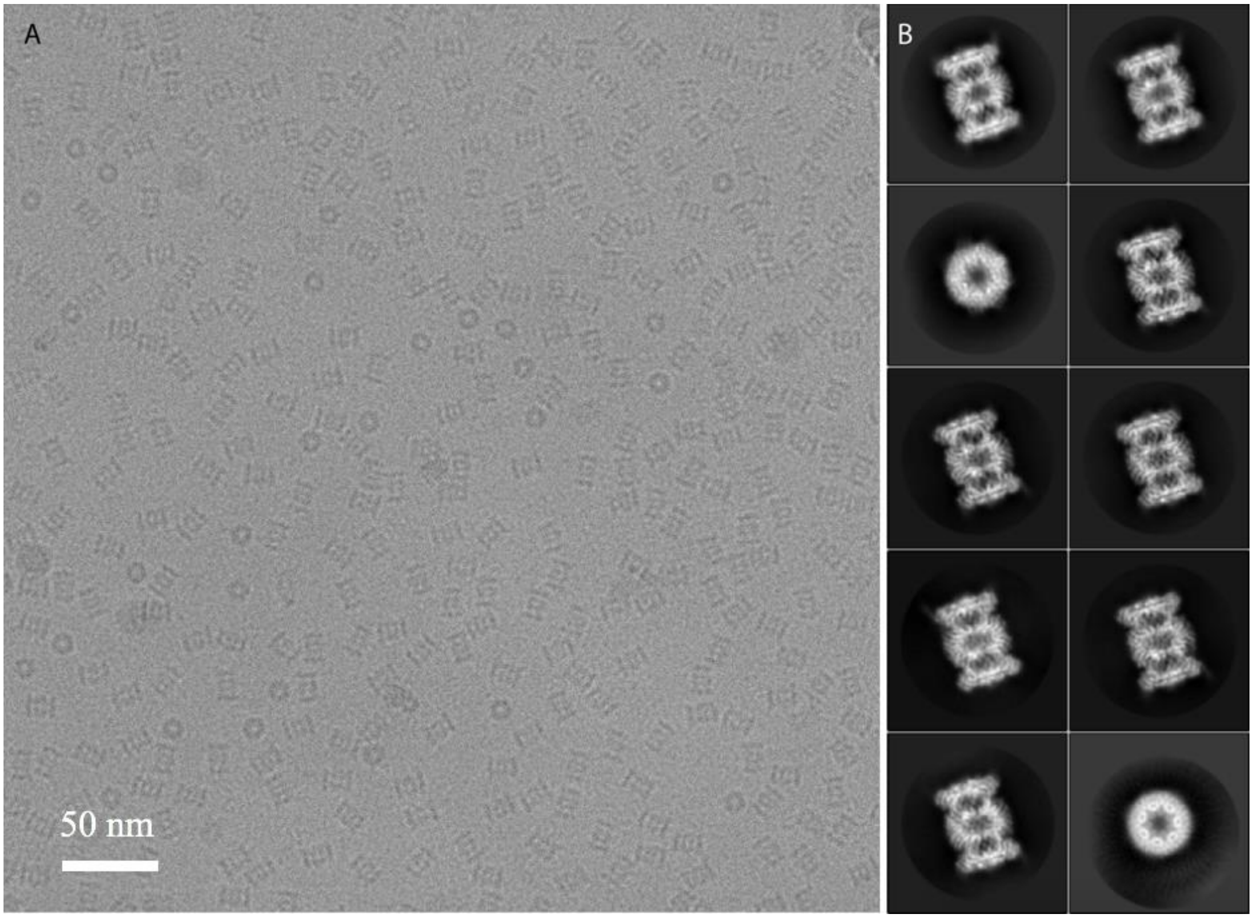

Supplementary Figure 2. Cryo-EM of the human i-20S incubated with the PKS21004. (**A**) A typical raw electron micrograph. A total of 2622 such micrographs were recorded with a Gatan K2 summit direct detector in a JEM-3200FS operated at a high tension of 300 kV. Both top and side views were present in the dataset. (**B**) Selected 2D class averages of the human core particles.

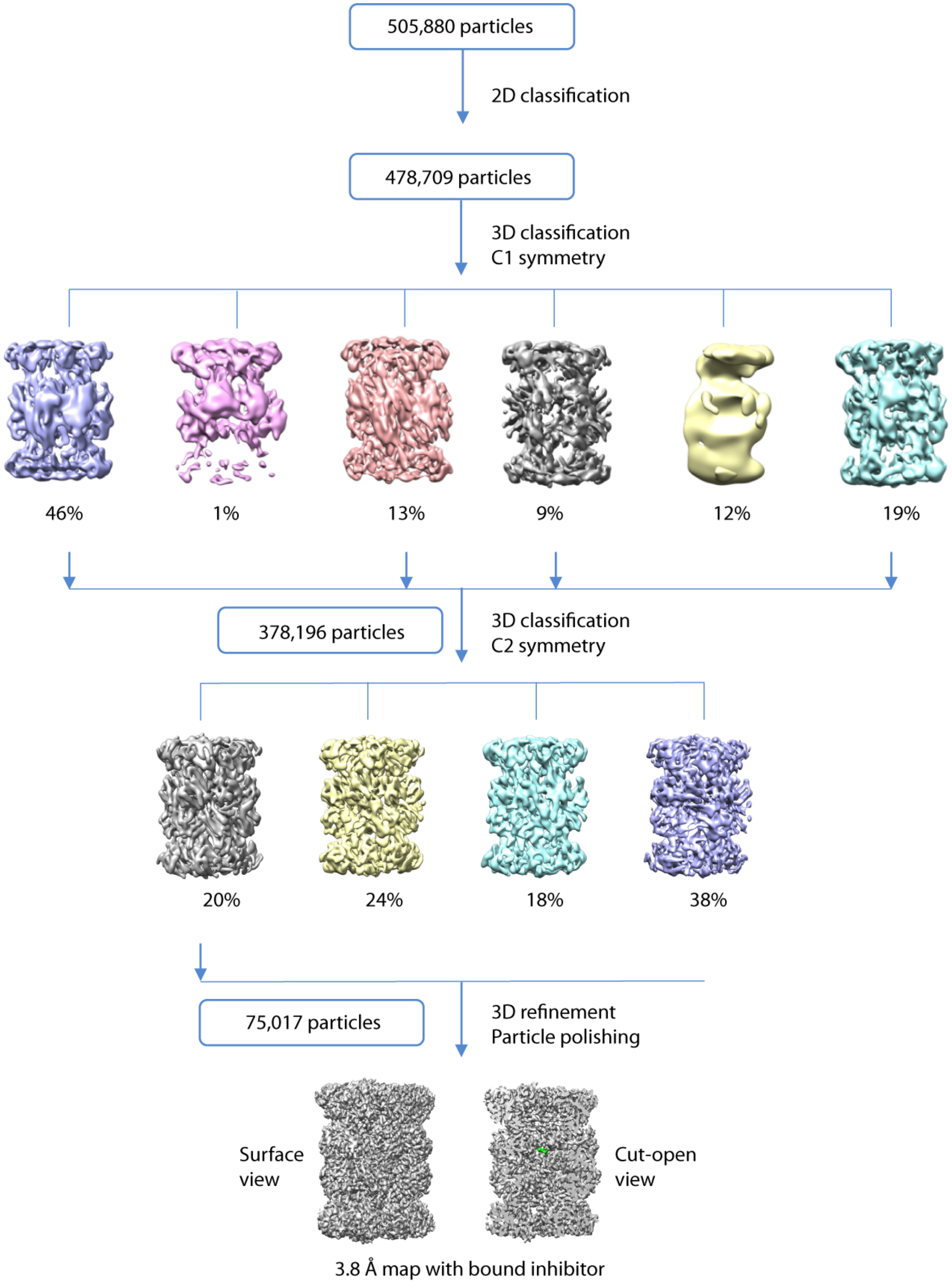
505,880 particles
2D classification
478,709 particles
3D classification
C1 symmetry
46%
1%
13%
9%
12%
19%
378,196 particles
3D classification
C2 symmetry
20%
24%
18%
38%
75,017 particles
3D refinement
Particle polishing
Surface view
Cut-open view
3.8 Å map with bound inhibitor

Supplementary Figure 3. A flow chart for 2D and 3D image classification and 3D refinement. A total of ~ 500,000 raw particles were selected from micrographs. After 3D sorting and 3D classification, only ~ 75,000 particles were used for final 3D reconstruction in RELION 2.0.

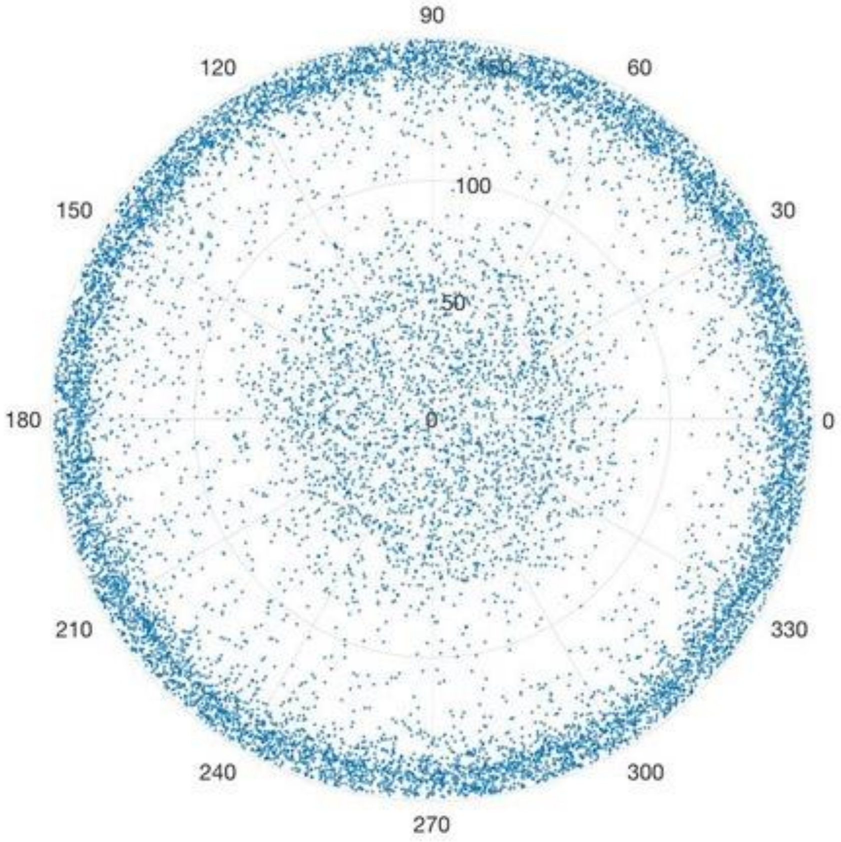

Supplementary Figure 4. Euler angle distribution of the human i-20S-PKS21004 particles.

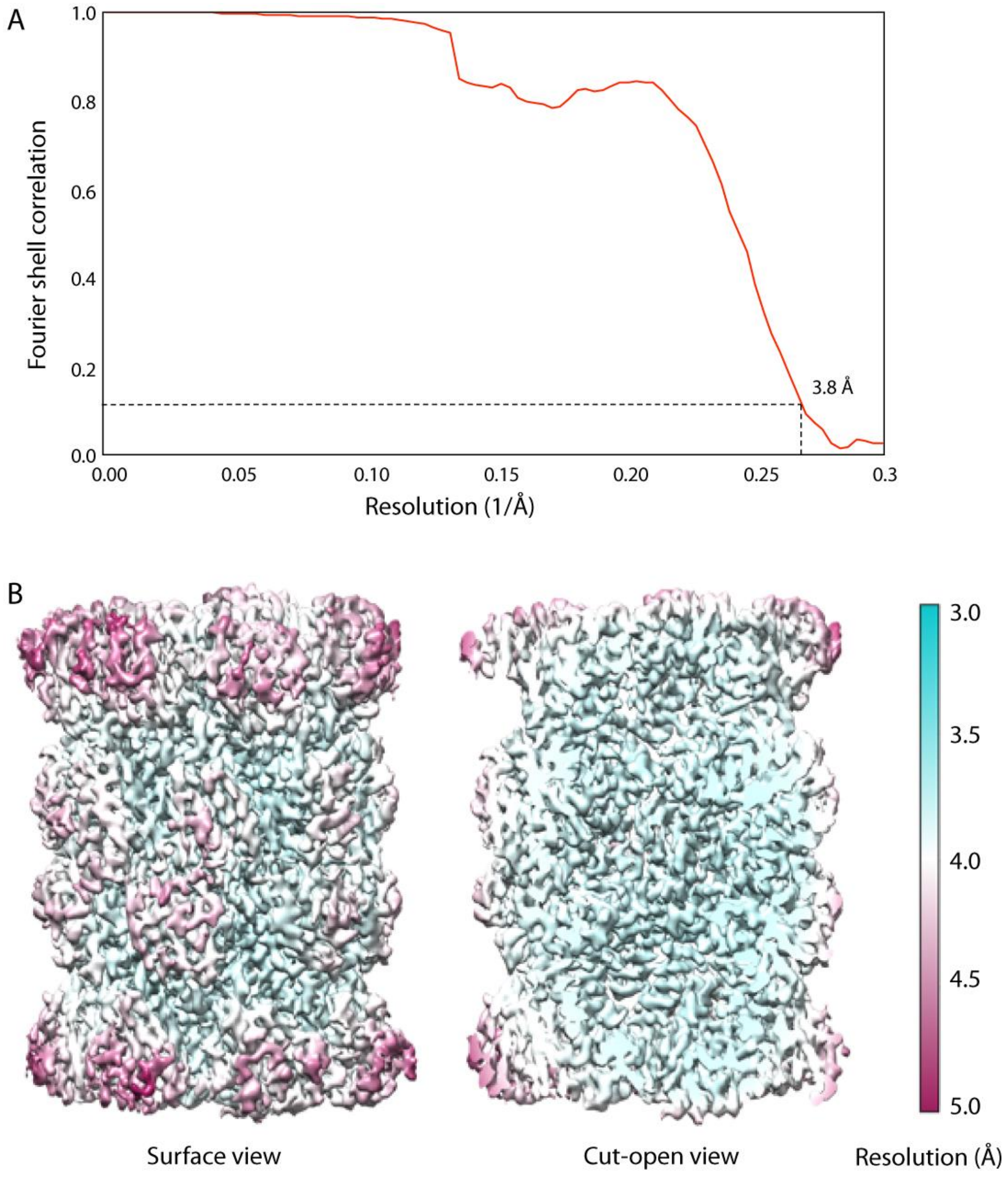

Supplementary Figure 5. Estimation of the resolution of the final 3D cryo-EM density map. (**A**) The gold-standard Fourier shell correlation suggests an average resolution of 3.8 Å at the 0.143 correlation threshold. (**B**) Local resolution map color-coded on the surface rendered view of the 3D map. Left: outer surface view; Right: Vertically cut-open view showing the better resolution in the interior of the core particle.

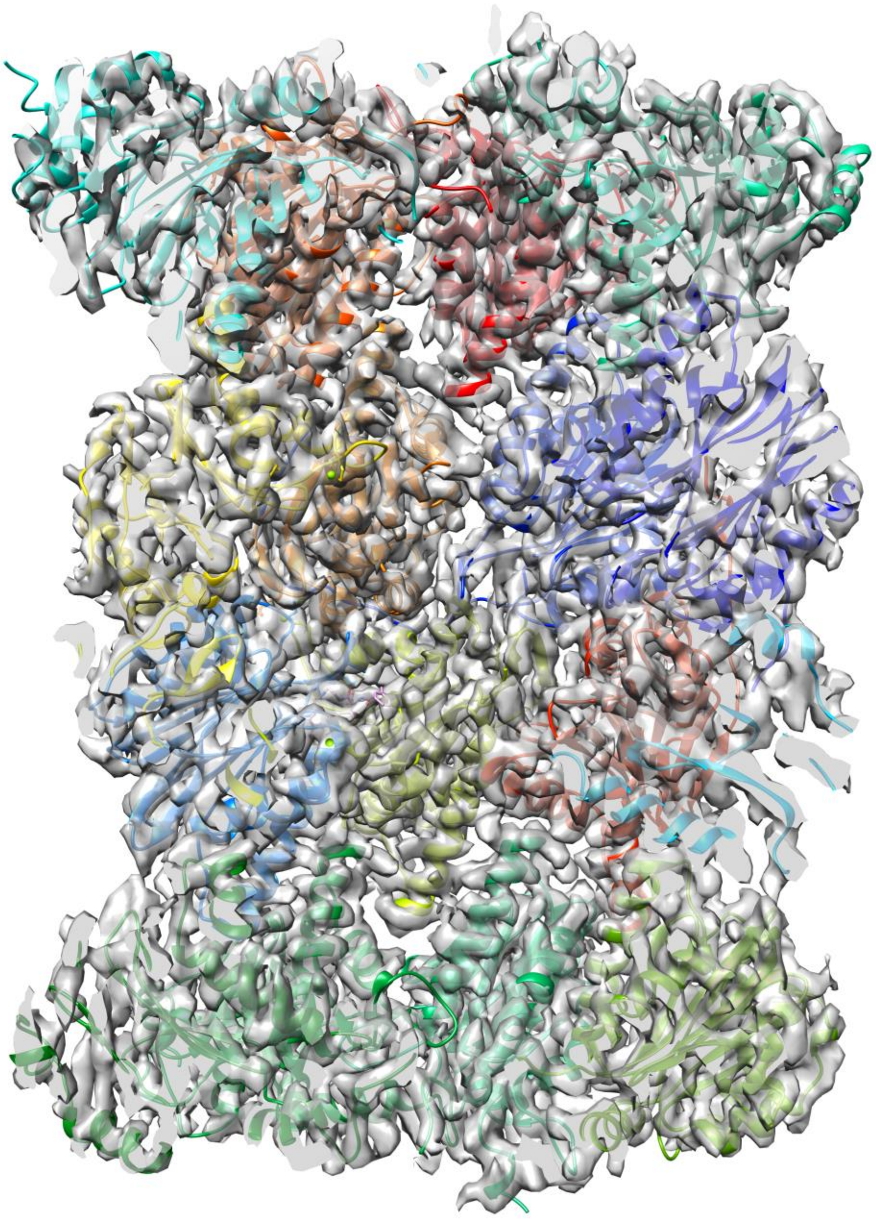

Supplementary Figure 6. **Cryo-EM 3D map of human i-20S is shown as a semi-transparent surface-rendered and cut-open view.** The atomic model is superimposed on the map and shown in cartoon.

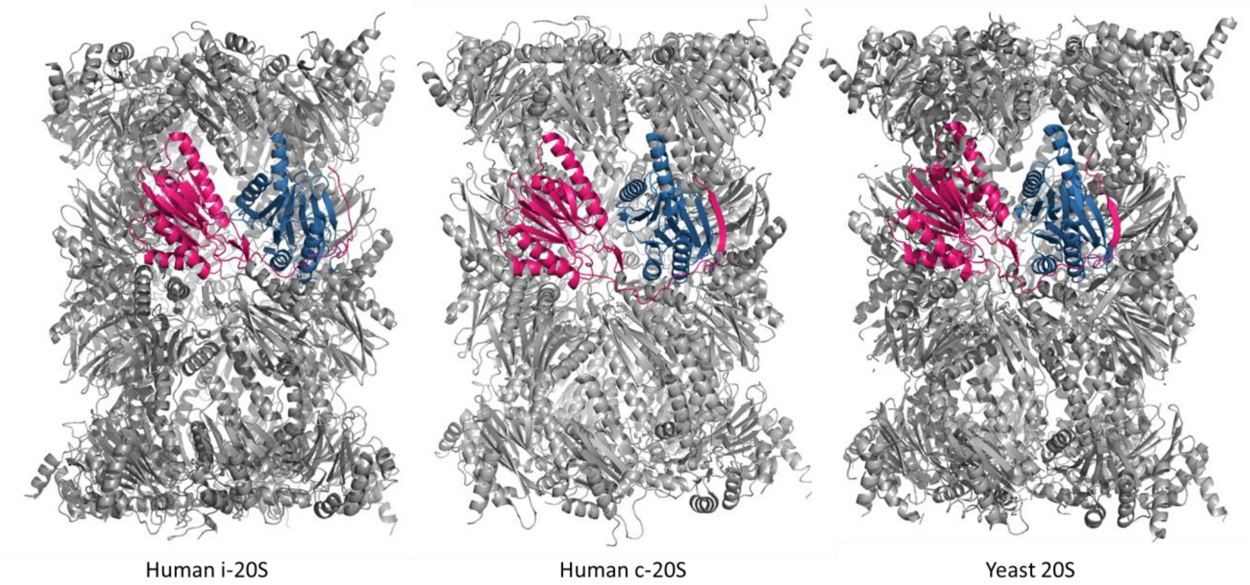

Supplementary Figure 7. **C-termini of the β2 (in red) subunit in yeast proteasome (2F16), human constitutive proteasome (4R3O) and human immunoproteasome embrace β3 (in blue) subunits, respectively.**

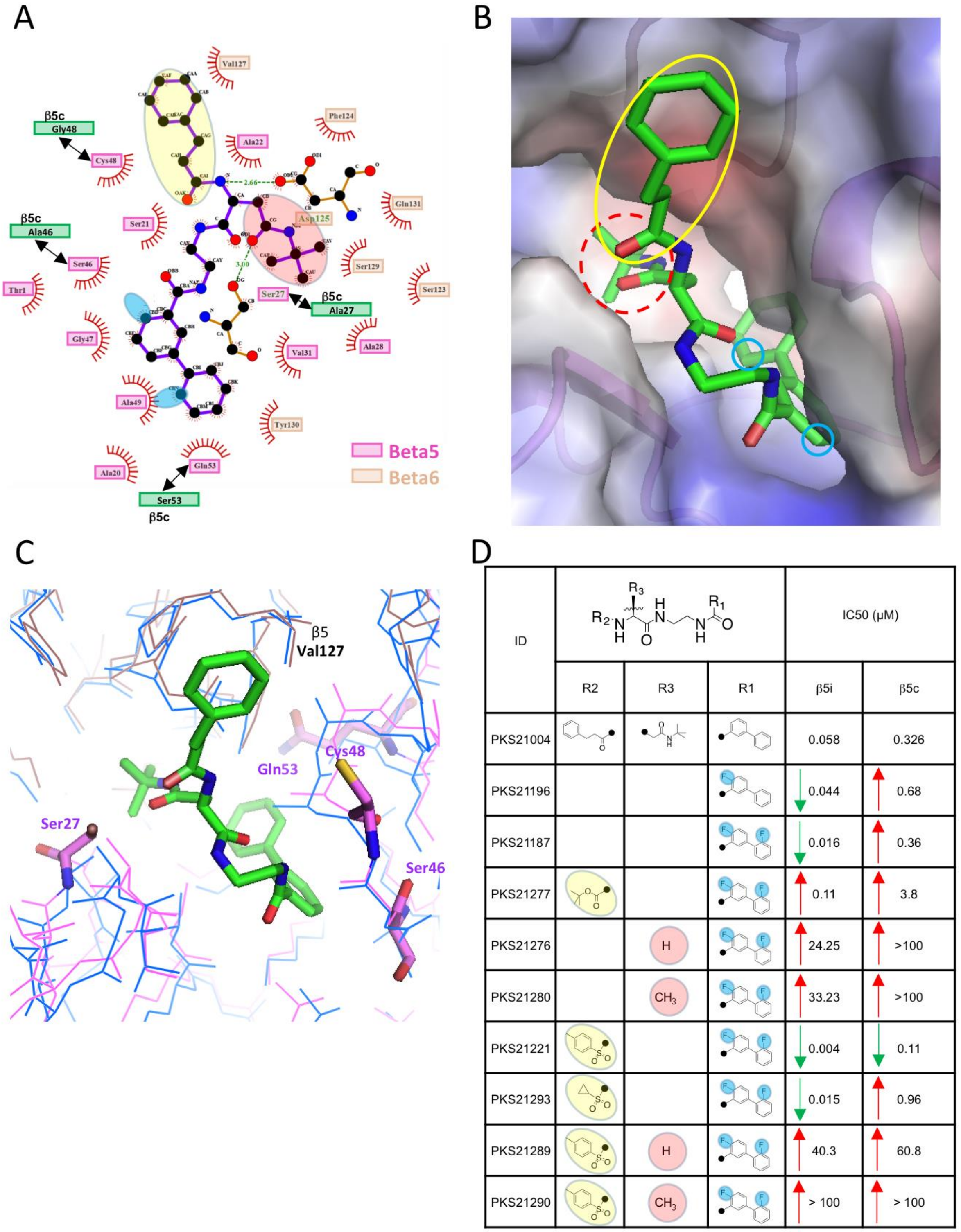

Supplementary Figure 8. SAR analysis of AsnEDA inhibition of the human i-20S. (**A**) Human i-20S-PKS21004 interaction map generated by Ligplot (http://www.ebi.ac.uk/thornton-

srv/software/LIGPLOT/). The circles of different colors highlight the PKS21004 sites modified for optimization of AsnEDA. The residues of β5i and β5c subunits involved in inhibitor binding and may account for selectivity are plotted in green. (**B**) The electrostatic potential surface presentation of the inhibitor PKS21004 binding pocket. (**C**) Alignment of the inhibitor binding pocket of human immuno-proteasome (β5i in magenta, β6i in brown, PKS21004 in green) with that of the constitutive proteasome (Blue, PDB code 5LF4). Four non-conserved residues near the inhibitor are labeled and shown in sticks. (**D**) Structures and IC_{50}s against human β5i and β5c of several optimized AsnEDAs. Red and green arrows mark the decreased or increased inhibition potency, respectively.

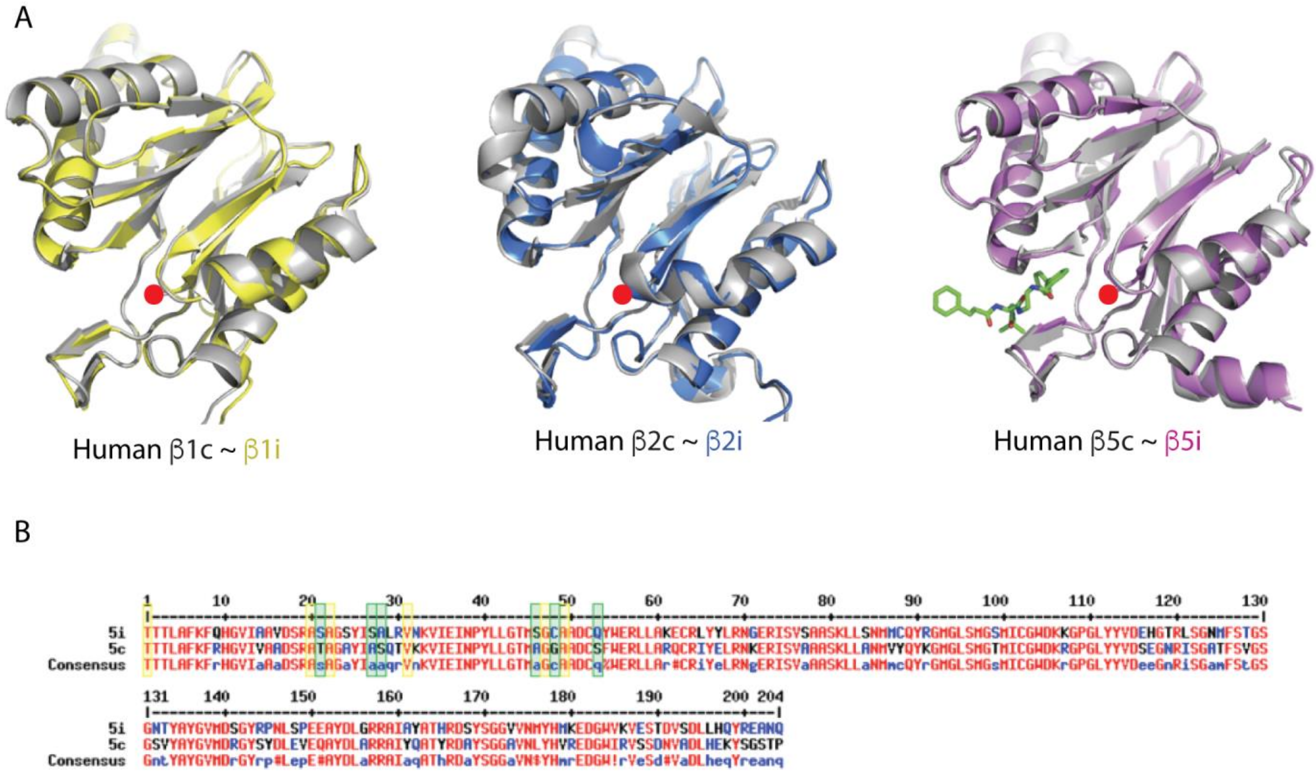

Supplementary Figure 9. Sequence and structural comparisons between the human immune- and constitutive proteasome subunits. (**A**) Alignment of the structures of β1, β2 and β5 subunits in human immuno-proteasome with the constitutive proteasome (PDB code 5LF1). Red dots denote the catalytic Thr1 of the β1 (Left), β2 (Middle) and β5 subunits (Right). The PKS21004 binding site in was shown in β5i as green sticks. (**B**) Sequence alignment of the β5i and β5c subunits of human proteasomes. Residues involved in inhibitor binding are highlighted by yellow (Consensus) or green (Variants) background.

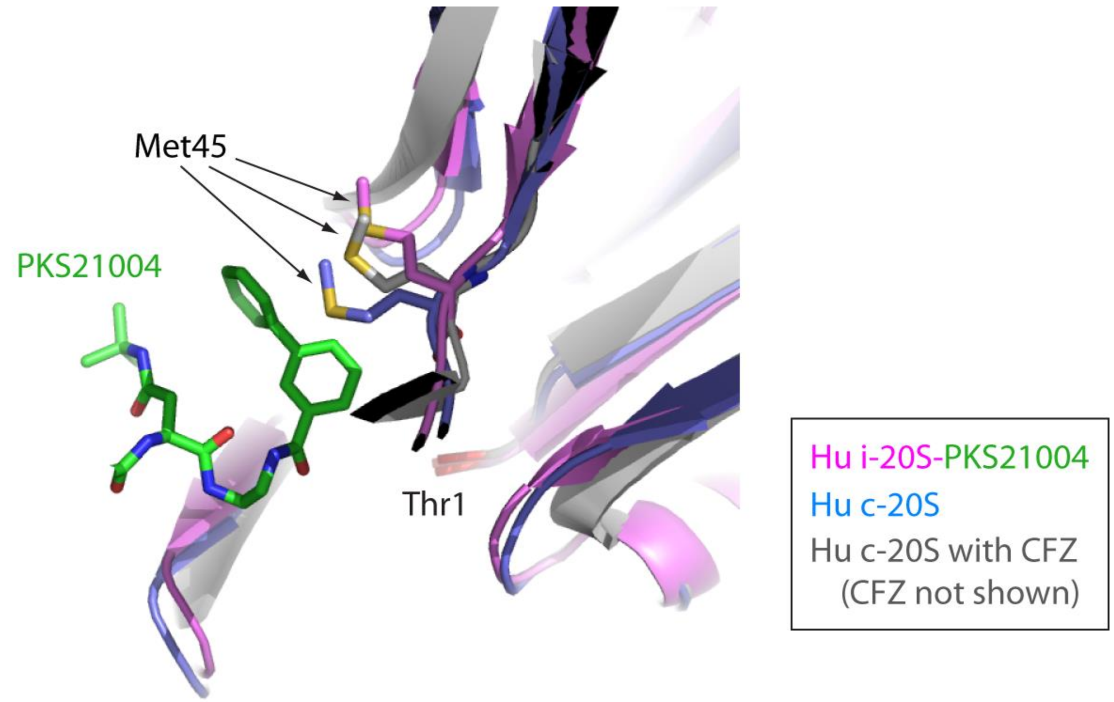

Supplementary Figure 10. **Superimposition of the interface regions of β5i-β6 with PKS21004 and of β5c-β6 of c-20S with and without carfilzomib** (Hu c-20S +/- CFZ, Harshbarger W et al, *Structure* **2015**, 418-24). Met45 appears to contribute to the selectivity of the PKS21004 against β5i over β5c. Met45 in c-20S clashes with the biphenyl moiety of the PKS21004. However, Met45 side chain in β5i is in the preformed optimal configuration that allows bulky moiety to bind.

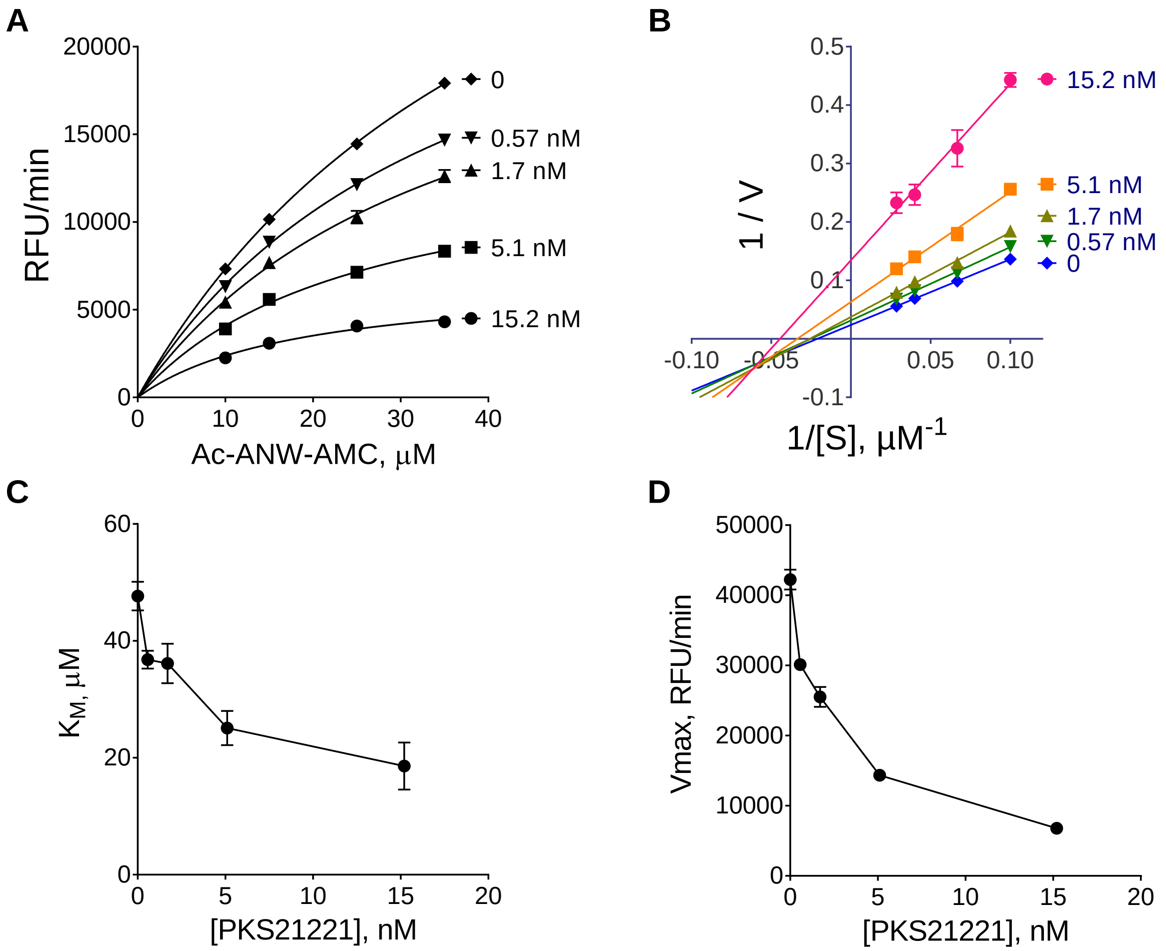

Supplementary Figure 11. **Inhibition kinetics of PKS21221 against i-20S β5i.** (**A**) Steady state velocities of the Ac-ANW-AMC hydrolysis at 10, 15, 25, 35 μM by i-20S β5i (0.4 nM) in the presence of PKS21221 at indicated concentrations were determined at 37 °C and plotted by RFU versus substrate concentration (A); values of K_M, K_i and α were estimated by PRISM using equation $V = (Vmax / (1 + I / (\alpha * K_i))) * S / (K_M * (1 + I / K_i) / (1 + I / (\alpha * K_i)) + S)$; (**B**) Lineweaver-Burk plot of the data in (A). (**C**) Plot of KM versus concentrations of PKS21221 indicates a mixed type inhibition. (**D**) Plot of Vmax versus concentrations of PKS21221.

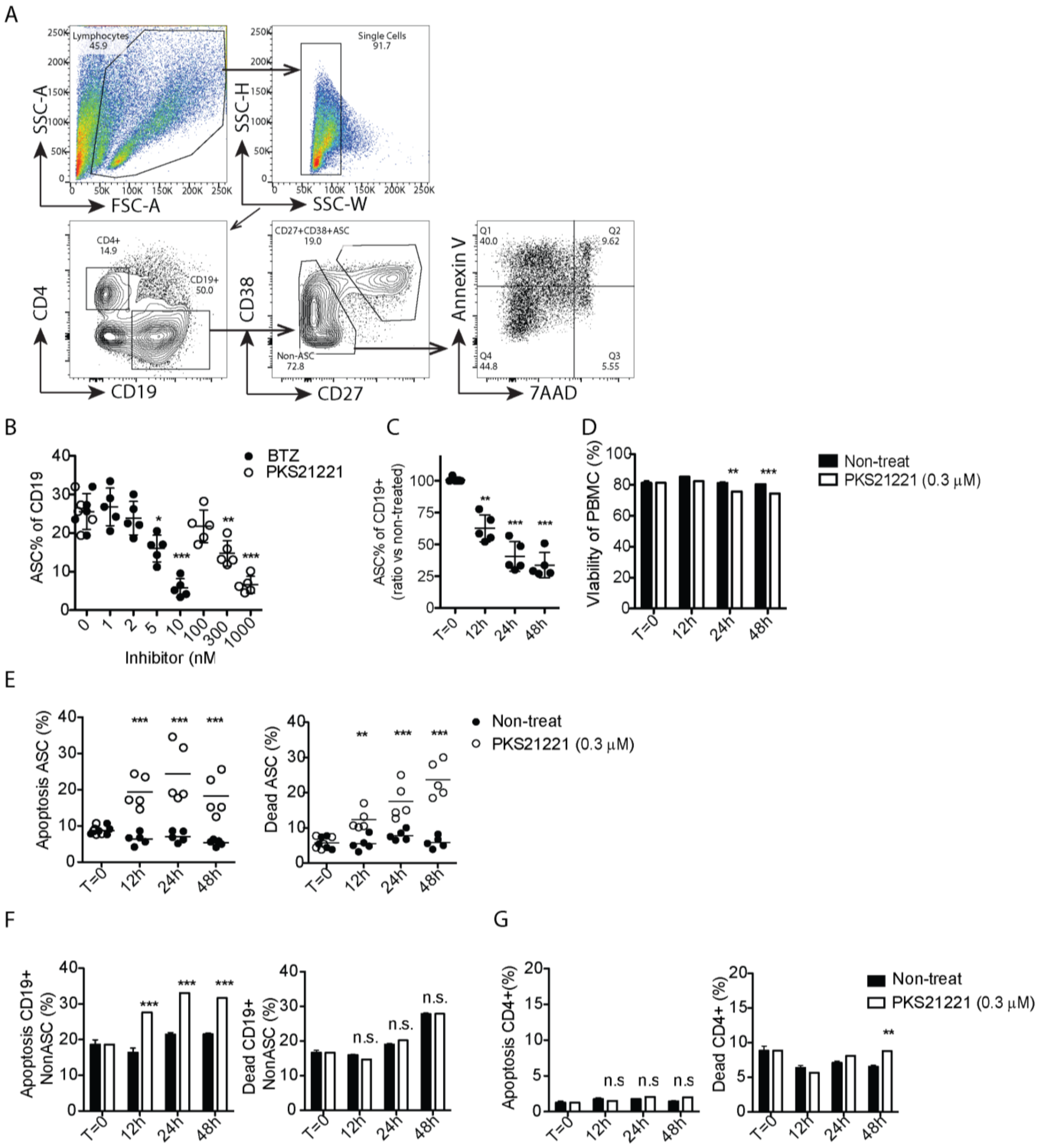

Supplementary Figure 12. (A) The gating strategy of flow cytometry (for Figure 5 and supplementary Figure S12). Single cells were gated based on FSC/SSC and SSC-H/SSC-W. ASC were

defined as $CD19^+CD27^+CD38^+$ cells. Apoptotic cells (Annexin V^+, $7AAD^-$) and dead cells (Annexin V^+, $7AAD^+$) were gated as shown in quadrants. (**B**) Effect of bortezomib (BTZ) and PKS21221 on ASC percentage in ASC differentiating culture. BTZ or PKS21221 were added for 12 hours on Day 5 ASC differentiation culture as described in Figure 5. Both compounds decreased the percentage of ASC in a dose-dependent manner. Each dot represents the result of a different healthy donor (n = 5), pooled from two independent experiments. (**C**) Percentage of ASC in $CD19^+$ cells treated with PKS21221 (300 nM). PKS21221 was added on Day 5 to ASC differentiation culture and incubated for different length of time. The values were normalized by those of non-treated control. PKS21221 decreased the percentage of ASC in a time-dependent manner. (**D**) Viability of total PBMCs during the culture were minimally affected by PKS21221 treatment. A representative data of one donor was shown (mean ± SEM of triplicate). (**E**) Apoptotic and dead ASC percentages after treatment for different length of time were shown. Each dot represents the result of a different healthy donor (n=5). Apoptotic and dead cell percentages in $CD19^+$Non-ASC (**F**) and $CD4^+$ populations (**G**) were shown. PKS21221 increased apoptosis of $CD19^+$Non-ASC but not that of $CD4^+$ cells. *$p<0.05$, **$p<0.01$, ***$p<0.001$, by paired *t*-test compared with non-treated group.

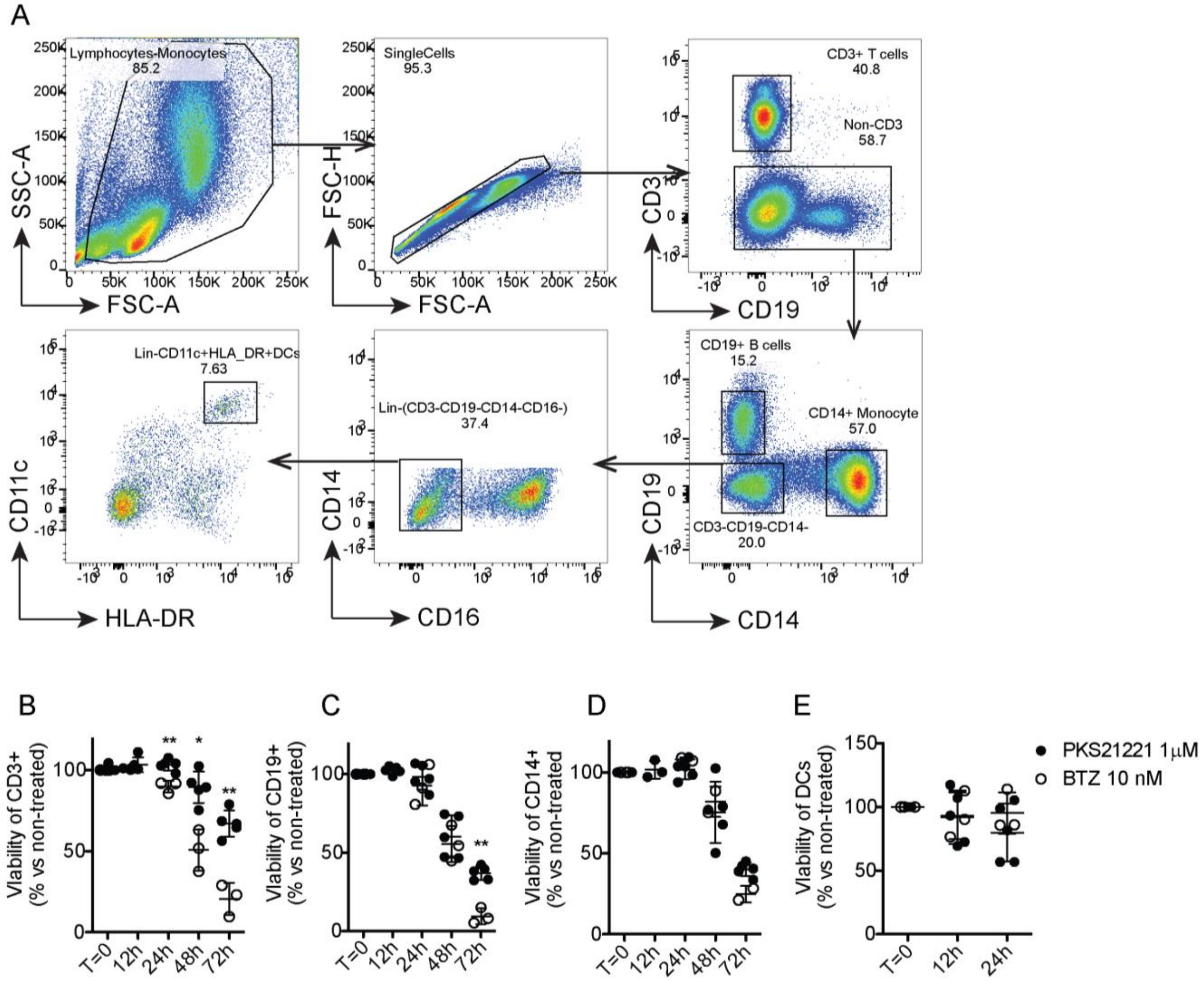

Supplementary Figure 13. Effect of PKS21221 and BTZ on the viability of PBMCs. PBMCs were cultured in the presence or absence of PKS21221 or BTZ for documented length of time and the viability of different populations was assessed by Fixable Viability Dye. (**A**) The gating strategy of flow cytometry. Single cells were gated based on FSC/SSC and FSC-H/FSC-A. T cells ($CD3^+$), B cells ($CD19^+CD3^-CD14^-$), monocytes ($CD14^+$), dendritic cells ($CD3^-CD19^-CD14^-CD16^-HLADR^+CD11c^+$) were defined as shown. Viability of T cells (**B**), B cells (**C**), monocytes (**D**), and dendritic cells (**E**) were shown. Viability of dendritic cells was assessed up to 24 hours incubation because the viability of dendritic cells in non-treated condition was lower than 10 % and could not be reliably analyzed. The values were normalized by those of non-treated control. Each dot represents the result of a different healthy donor (n = 5, for PKS21221, n=3 for BTZ)* $p < 0.05$, ** $p < 0.01$, *** $p < 0.001$, by paired *t*-test, BTZ-treated group was compared with PKS21221-treated group.

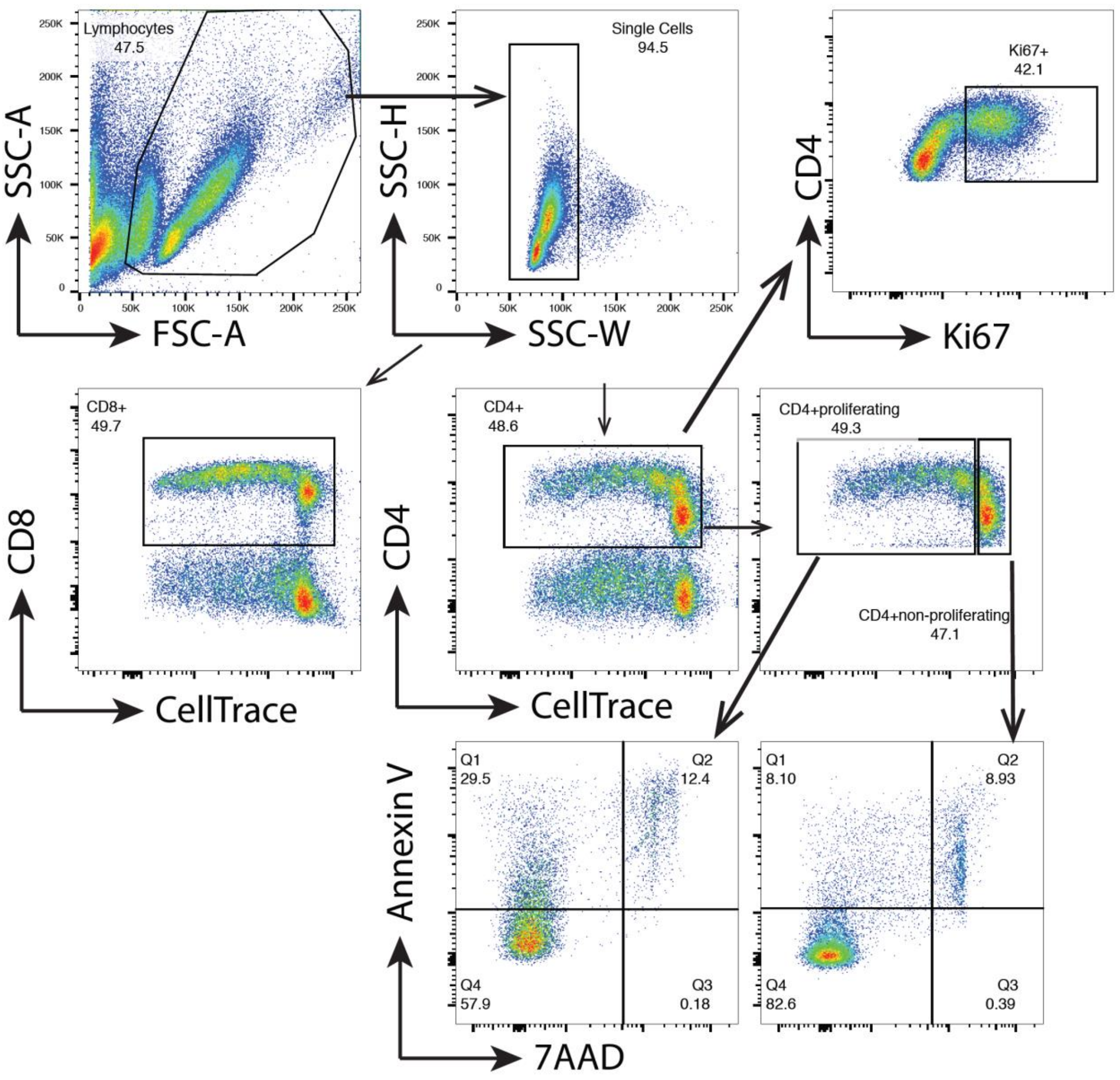

Supplementary Figure 14. The Gating strategy of flow cytometry in Figure 6. Single cells were gated based on FSC/SSC and SSC-H/SSC-W. Cell proliferation was assessed by dilution of CellTrace dye and Ki67 staining. Apoptosis and viability of the proliferating or non-proliferating cells were assessed by Annexin V and 7-AAD.

Supplementary Table 1. IC50s of compounds against β1i, β2i, β1c and β2c of human immuno- and constitutive proteasomes

	ID	IC50 (µM)			
		β1i - PAL	β1c - LLE	β2i - VLR	β2c - VLR
1	PKS3080	> 50	> 50	> 50	> 50
2	PKS21025	> 50	> 50	> 50	> 50
3	PKS21003	> 50	> 50	> 50	> 50
4	PKS21004	> 50	> 50	> 50	> 50
5	PKS21018	> 50	> 50	> 50	> 50
6	PKS21019	> 50	> 50	> 50	> 50
7	PKS21026	> 50	> 50	> 50	> 50
8	PKS21028	> 50	> 50	> 50	> 50
9	PKS21196	> 50	> 50	> 50	> 50
10	PKS21195	> 50	> 50	> 50	> 50
11	PKS21187	> 50	> 50	> 50	> 50
12	PKS21277	> 50	> 50	> 50	> 50
13	PKS21276	> 50	> 50	> 50	> 50
14	PKS21280	> 50	> 50	> 50	> 50
15	PKS21281	> 50	> 50	> 50	> 50
16	PKS21284	> 50	> 50	> 50	> 50
17	PKS21221	> 50	> 50	> 50	> 50
18	PKS21289	> 50	> 50	> 50	> 50
19	PKS21290	> 50	> 50	> 50	> 50
20	PKS21288	> 50	> 50	> 50	> 50
21	PKS21208	> 50	> 50	> 50	> 50
22	PKS21294	> 50	> 50	> 50	> 50
23	PKS21293	> 50	> 50	> 50	> 50

PAL: Ac-PAL-AMC; LLE: Z-LLE-AMC; VLR: Z-VLR-AMC

Supplementary Methods

Synthesis

Chemicals and Spectroscopy: Unless otherwise stated, all commercially available materials were purchased from Bachem, Aldrich, P3 BioSystems, or other vendors and were used as received. All non-aqueous reactions were performed under argon in oven-dried glasswares. Routine monitoring of reactions was performed using Waters Acquity Ultra Performance Liquid Chromatography (UPLC). All HPLC purifications were done by Varian PrepStar HPLC system or Waters Autopure (mass directed purification system) using Prep C18 5μm OBD (19 X 150 mm) column. ^{1}H- and ^{13}C- NMR spectra were acquired on a Bruker DRX-500 spectrometer. Chemical shifts δ are expressed in parts per million, with the solvent resonance as an internal standard (chloroform-*d*, ^{1}H: 7.26; ^{13}C: 77.16 ppm; DMSO-*d6*, ^{1}H: 2.50 ppm; ^{13}C: 39.52 ppm). Hexafluorobenzene was used as internal standard for ^{19}F NMR. NMR data are reported as following: chemical shift, multiplicity (s = singlet, d = doublet, t = triplet, q = quartet, m = multiplet, br = broad), coupling constant, and integration.

General Procedure for HATU mediated amide bond formation: The carboxylic acid (1 equivalent), *O*-(7-azabenzotriazole-1-yl)-*N*,*N*,*N*,*N*'-tetramethyluronium hexafluorophosphate (HATU, 1.2 equivalent) and 1-hydroxy-7-azabenzotriazole (HOAt) 0.6 M in DMF (1 equivalent) were dissolved in DMF. The solution was cooled to 0 °C using ice bath. Amine (1 equivalent) and Hünig base (2 - 3 equiv) were added to the reaction mixture at 0 °C. The reaction mixture was stirred at 0 °C. After completion of reaction (2 – 3 hours; monitored by UPLC), water was added to the reaction mixture and mixture was stirred for 15 minutes. The product was isolated by either filtration or ethyl acetate extraction.

General Procedure for Boc-Deprotection: The substrate was dissolved in dichloromethane and the solution was cooled to 0 °C. Trifluoroacetic acid (20% v/v with respect to dichloromethane) was added to the solution drop wise at 0 °C with constant stirring. The mixture was allowed to warm to room temperature slowly (over a period of 1 hour), and stirred until the completion of reaction (3 – 4 hours; monitored by UPLC). Excess trifluoroacetic acid and dichloromethane were evaporated and crude was dried under vacuum.

General Procedure for *O*-Debenzylation: The substrate was dissolved in methanol (or THF). Palladium on carbon (10%) was added carefully. Residual air from the flask was removed and flask was flushed with hydrogen. The mixture was stirred at room temperature for 3 – 4 hours under hydrogen atmosphere using a hydrogen balloon. After completion of reaction, the mixture was filtered through celite. Filtrate was evaporated and dried under vacuum to give product.

General Procedure for *N*-Sulfonamide Preparation of Amines: The primary amine (generally TFA salt) was dissolved in dichloromethane. The solution was cooled to 0 °C and triethylamine (2.0 – 3.0 eq.) was added. Sulfonyl chloride (1.5 eq.) was added to the solution in one portion and reaction mixture was warmed to room temperature (over 15 minutes). After completion of reaction (2 - 3 hours), dichloromethane was evaporated and crude product was isolated by ethyl acetate extraction.

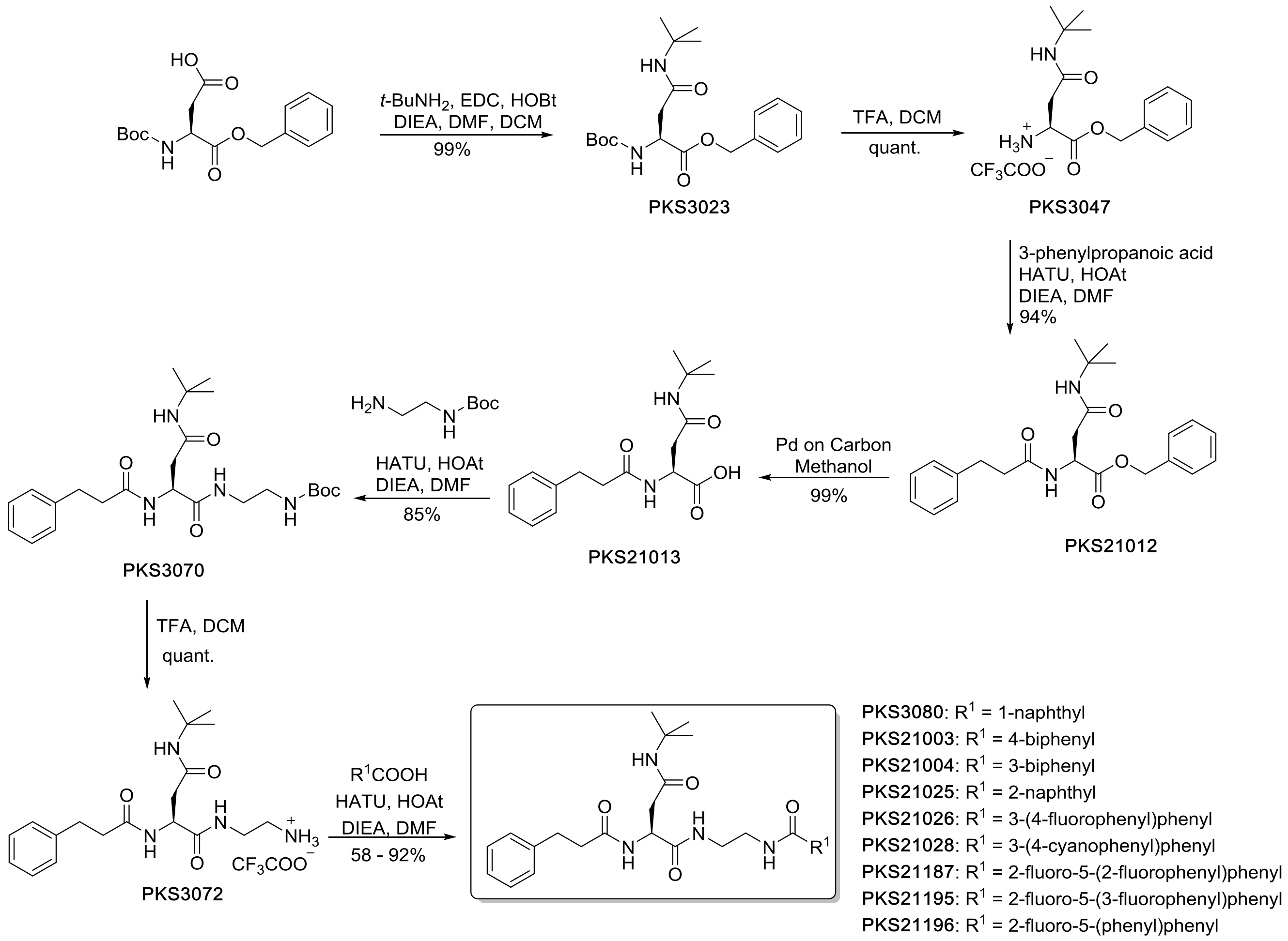

Scheme 1

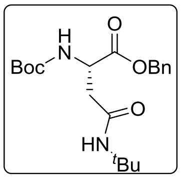

PKS3023: Boc-Asp-OBn (5.01 g, 15.49 mmol) and *N*-(3-dimethylaminopropyl)-*N′*-ethylcarbodiimide hydrochloride (EDC) (3.56 g, 18.59 mmol) were dissolved in dichloromethane (75.00 mL) under argon atmosphere. The solution was cooled to 0 °C and a solution of 1-hydroxybenzotriazole (2.30 g, 17.04 mmol) in DMF (10.00 mL) was added to the mixture. After stirring for 10 minutes at 0 °C, Hünig's base (4.01 g, 30.99 mmol) and *tert*-butylamine (1.70 g, 23.24 mmol) were added. Reaction mixture was allowed to warm to room temperature (23 °C) slowly and stirred at room temperature overnight. After completion of reaction, dichloromethane was evaporated and crude was diluted with water. Mixture was extracted twice with ethyl acetate. Combined organic layer was washed with aq. $NaHCO_3$, water, 1N HCl, water followed by saturated brine solution. Ethyl acetate layer was dried over anhydrous Na_2SO_4 and evaporated to give product (5.80 g, 99%) as white solid. The product was used in next step without further purification. ^{1}H NMR (500 MHz, Chloroform-*d*) δ 7.36 – 7.26 (m, 5H), 5.92 – 5.76 (m, 1H), 5.41 (br, 1H), 5.21 (d, *J* = 12.4 Hz, 1H), 5.14 (d, *J* = 12.4 Hz, 1H), 4.57 – 4.42 (m, 1H), 2.79 (dd, *J* = 15.8, 4.9 Hz, 1H), 2.62 (dd, *J* = 15.8, 4.2 Hz, 1H), 1.42 (s, 9H), 1.29 (s, 9H). ES$^+$ calc. for $C_{20}H_{31}N_2O_5$ $[M+H]^+$: 379.2. Found: 379.2

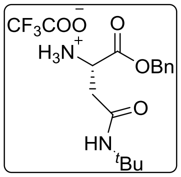

PKS3047: The title compound was synthesized by following the general procedure for boc-deprotection of **PKS3023** (3.84 g, 10.15 mmol). After completion of reaction (3h), excess trifluoroacetic acid and dichloromethane were evaporated. Crude was dried under vacuum to give product (3.98 g, quant.) as colorless paste. Product was used in next step without further purification. ^{1}H NMR (500 MHz, DMSO-*d*$_6$) δ 8.36 (br, 3H), 7.85 (s, 1H), 7.46 – 7.29 (m, 5H), 5.24 (d, *J* = 12.6 Hz, 1H), 5.18 (d, *J* = 12.6 Hz, 1H), 4.39 – 4.29 (m, 1H), 2.83 – 2.66 (m, 2H), 1.22 (s, 9H). ES$^+$ calc. for $C_{15}H_{23}N_2O_3$ $[M+H]^+$: 279.2. Found: 279.2

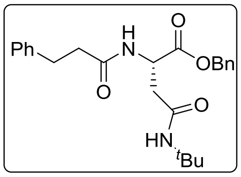

PKS21012: The title compound was synthesized by following the general procedure for the HATU mediated coupling of 3-phenylpropanoic acid (1.68 g, 11.17 mmol) with **PKS3047** (3.98 g, 10.15 mmol). After completion of reaction (3 h), water was added. The white precipitate appeared, was filtered and washed with water. Precipitate was dried in air to give product (3.92 g, 94%) as white solid. ^{1}H NMR (500 MHz, Chloroform-*d*) δ 7.39 – 7.29 (m, 5H), 7.29 – 7.23 (m, 2H), 7.21 – 7.14 (m, 3H), 6.88 (d, *J* = 8.0 Hz, 1H), 5.32 (br, 1H), 5.20 (d, *J* = 12.3 Hz, 1H), 5.14 (d, *J* = 12.3 Hz, 1H), 4.84 – 4.77 (m, 1H), 2.95 (t, *J* = 7.9

Hz, 2H), 2.81 (dd, *J* = 15.7, 4.4 Hz, 1H), 2.61 – 2.47 (m, 3H), 1.28 (s, 9H). ES$^+$ calc. for $C_{24}H_{31}N_2O_4$ [M+H]$^+$: 411.2. Found: 411.3

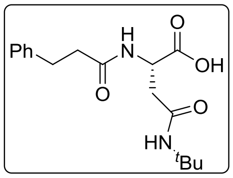

PKS21013: The title compound was synthesized by following the general procedure for *O*-debenzylation of **PKS21012** (1.44 g, 3.50 mmol) in methanol (17.00 mL). The procedure yielded the product (1.11 g, 99%) as white solid. Product was used in next step without further purification. ^{1}H NMR (500 MHz, DMSO-d_6) δ 12.51 (br, 1H), 8.05 (d, *J* = 7.9 Hz, 1H), 7.43 (s, 1H), 7.29 – 7.23 (m, 2H), 7.22 – 7.14 (m, 3H), 4.55 – 4.42 (m, 1H), 2.82 – 2.77 (m, 2H), 2.52 – 2.45 (m, 1H), 2.42 – 2.35 (m, 3H), 1.22 (s, 9H). ES$^+$ calc. for $C_{17}H_{25}N_2O_4$ [M+H]$^+$: 321.2. Found: 321.3

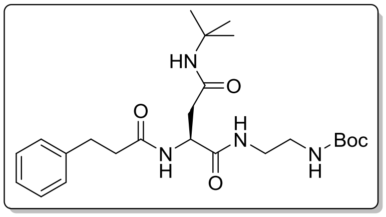

PKS3070: The title compound was synthesized by following the general procedure of HATU mediated coupling of **PKS21013** (778 mg, 2.43 mmol) and *N*-Boc-ethylenediamine (428 mg, 2.67 mmol). After completion of reaction (1.5 h), water was added. The white precipitate formed, was filtered and dried in air to give product (955 mg, 85%). Product was used in next step without further purification. ^{1}H NMR (500 MHz, Chloroform-*d*) δ 7.32 – 7.27 (m, 3H), 7.24 – 7.18 (m, 3H), 6.92 (br, 1H), 5.72 (br, 1H), 5.01 (br, 1H), 4.65 – 4.56 (m, 1H), 3.29 – 3.14 (m, 4H), 3.05 – 2.92 (m, 2H), 2.70 (dd, *J* = 15.0, 3.7 Hz, 1H), 2.60 (t, *J* = 7.6 Hz, 2H), 2.31 (dd, *J* = 15.0, 6.1 Hz, 1H), 1.44 (s, 9H), 1.31 (s, 9H). ES$^+$ calc. for $C_{24}H_{39}N_4O_5$ [M+H]$^+$: 463.3. Found: 463.4

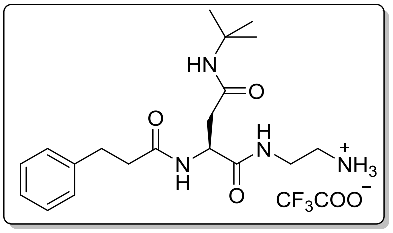

PKS3072: The title compound was synthesized by following the general procedure for Boc-deprotection of **PKS3070** (953 mg, 2.06 mmol). Isolated crude was dried and triturated with diethyl ether to give a white solid. Diethyl ether was decanted and white solid was dried under vacuum to give product (980 mg, quant.). Product was used in next step without further purification. ^{1}H NMR (500 MHz, DMSO-d_6) δ 8.08 (d, *J* = 7.9 Hz, 1H), 8.05 (t, *J* = 5.6 Hz, 1H), 7.80 (br, 3H), 7.56 (s, 1H), 7.30 – 7.23 (m, 2H), 7.22 – 7.14 (m, 3H), 4.50 – 4.42 (m, 1H), 3.42 – 3.31 (m, 1H), 3.29 – 3.19 (m, 1H), 2.92 – 2.82 (m, 2H), 2.80 (t, *J* = 7.9 Hz, 2H), 2.48 – 2.34 (m, 4H), 1.22 (s, 9H). ES$^+$ calc. for $C_{19}H_{31}N_4O_3$ [M+H]$^+$: 363.2. Found: 363.3

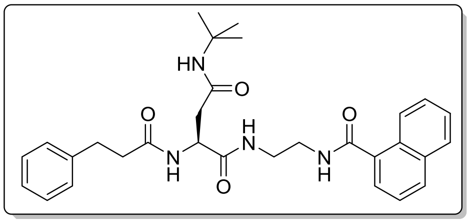

PKS3080: The title compound was synthesized by following the general procedure for HATU mediated coupling of 1-naphthoic acid (20.7 mg, 0.12 mmol) and **PKS3072** (47.6 mg, 0.1 mmol). After completion of reaction (1 h), mixture was purified by HPLC to give product (30.2 mg, 58%) as white solid. ^{1}H NMR (500 MHz, DMSO-d_6) δ 8.47 (t, *J* = 5.6 Hz, 1H), 8.25 – 8.18 (m, 1H), 8.03 – 7.92 (m, 4H), 7.66 (dd, *J* = 7.1, 1.3 Hz, 1H), 7.60 – 7.48 (m, 3H), 7.38 (s, 1H), 7.28 – 7.22 (m, 2H), 7.19 – 7.12 (m, 3H), 4.57 – 4.46 (m, 1H), 3.45 – 3.37 (m, 2H), 3.32 – 3.21 (m, 2H), 2.77 (t, *J* = 8.0 Hz, 2H), 2.46 (dd, *J* = 14.6, 6.0 Hz, 1H), 2.43 – 2.37 (m, 2H), 2.32 (dd, *J* = 14.6, 7.9 Hz, 1H), 1.19 (s, 9H). ^{13}C NMR (126 MHz, DMSO-d_6) δ 171.3, 171.2, 168.9, 168.8, 141.3, 134.7, 133.1, 129.8, 129.7, 128.3, 128.2, 128.1, 126.7, 126.2, 125.8, 125.5, 125.3, 124.9, 50.2, 50.0, 38.9, 38.7, 38.7, 36.9, 30.9, 28.4. HRMS calc. for $C_{30}H_{36}N_4O_4Na$ [M+Na]$^+$: 539.2634. Found: 539.2637.

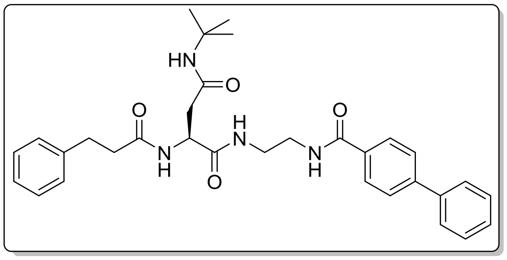

PKS21003: The title compound was synthesized by HATU mediated coupling of 4-phenylbenzoic acid (104.0 mg, 525 μmol) and **PKS3072** (250.0 mg, 525 μmol). After completion of reaction (1 h), water was added. The white precipitate obtained was filtered, washed with water and dried in air to give 284.0 mg white solid. The white solid was triturated with ethyl acetate and isolated by centrifugation (4255 X g, 10 min). Isolated white solid (235 mg, 82%) was pure product (by UPLC & NMR). ^{1}H NMR (500 MHz, DMSO-d_6) δ 8.51 – 8.44 (m, 1H), 8.00 (d, *J* = 7.9 Hz, 1H), 7.99 – 7.89 (m, 3H), 7.76 – 7.72 (m, 2H), 7.72 – 7.67 (m, 2H), 7.52 – 7.45 (m, 2H), 7.43 – 7.36 (m, 2H), 7.28 – 7.22 (m, 2H), 7.20 – 7.12 (m, 3H), 4.54 – 4.45 (m, 1H), 3.36 - 3.33 (m, 2H), 3.29 – 3.14 (m, 2H), 2.78 (t, *J* = 8.0 Hz, 2H), 2.48 – 2.38 (m, 3H), 2.32 (dd, *J* = 15.0, 8.0 Hz, 1H), 1.21 (s, 9H); ^{13}C NMR (126 MHz, DMSO) δ 171.3, 171.2, 168.9, 166.1, 142.7, 141.3, 139.2, 133.3, 129.0, 128.3, 128.1, 128.0, 127.9, 126.9, 126.5, 125.9, 50.2, 50.1, 38.9, 38.7, 38.7, 36.9, 31.0, 28.5. HRMS calc. for $C_{32}H_{38}N_4O_4Na$ [M+Na]$^+$: 565.2791. Found: 565.2786

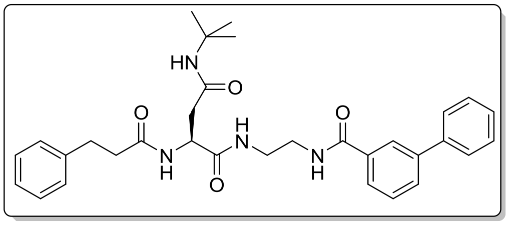

PKS21004: The title compound was synthesized by HATU mediated coupling of 3-phenylbenzoic acid (396.8 mg, 2.00 mmol) and **PKS3072** (867.2 mg, 1.82 mmol). After completion of reaction (2 h), water was added to the reaction mixture. An off white precipitate appeared. Precipitate was filtered and recrystallized from ethanol to give pure product (905 mg, 92%) as white solid. ^{1}H NMR (500 MHz, DMSO-*d6*) δ 8.57 (t, *J* = 5.5 Hz, 1H), 8.13 (s, 1H), 8.01 – 7.93 (m, 2H), 7.87 – 7.79 (m, 2H), 7.76 – 7.70 (m, 2H), 7.54 (t, *J* = 7.7 Hz, 1H), 7.51 – 7.46 (m, 2H), 7.43 – 7.35 (m, 2H), 7.28 – 7.22 (m, 2H), 7.19 – 7.13 (m, 3H), 4.54 – 4.46 (m, 1H), 3.38 – 3.15 (m, 4H), 2.77 (t, *J* = 7.9 Hz, 2H), 2.48 – 2.36 (m, 3H), 2.32 (dd, *J* = 14.7, 7.9 Hz, 1H), 1.20 (s, 9H); ^{13}C NMR (126 MHz, DMSO) δ 171.3, 171.2, 168.9, 166.3, 141.3, 140.2, 139.6, 135.1, 129.3, 128.9, 129.0, 128.3, 128.1, 127.7, 126.8, 126.4, 125.8, 125.4, 50.2, 50.0, 38.9, 38.7, 38.6, 36.9, 30.9, 28.4. HRMS calc. for $C_{32}H_{38}N_4O_4Na$ $[M+Na]^+$: 565.2791. Found: 565.2774

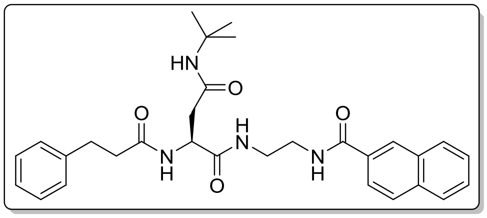

PKS21025: The title compound was synthesized by following the general procedure for HATU mediated coupling of 2-naphthoic acid (5.2 mg, 30 μmol) and **PKS3072** (11.9 mg, 25 μmol). After completion of reaction (1 h), mixture was purified by HPLC to give product (11.5 mg, 89%) as white solid. ^{1}H NMR (500 MHz, DMSO-*d6*) δ 8.61 (t, *J* = 5.6 Hz, 1H), 8.43 (s, 1H), 8.03 – 7.95 (m, 5H), 7.92 (d, *J* = 8.6 Hz, 1H), 7.65 – 7.53 (m, 2H), 7.40 (s, 1H), 7.29 – 7.20 (m, 2H), 7.20 – 7.10 (m, 3H), 4.55 – 4.46 (m, 1H), 3.44 – 3.34 (m, 2H), 3.32 – 3.18 (m, 2H), 2.77 (t, *J* = 8.0 Hz, 2H), 2.49 – 2.37 (m, 3H), 2.32 (dd, *J* = 14.6, 7.9 Hz, 1H), 1.19 (s, 9H); ^{13}C NMR (126 MHz, DMSO) δ 171.6, 171.6, 169.1, 166.9, 141.4, 134.3, 132.3, 131.9, 129.0, 128.5, 128.3, 128.0, 127.8, 127.6, 126.9, 126.1, 124.3, 50.4, 50.3, 39.2, 38.9, 38.8, 37.1, 31.1, 28.6. HRMS calc. for $C_{30}H_{36}N_4O_4Na$ $[M+Na]^+$: 539.2634. Found: 539.2617

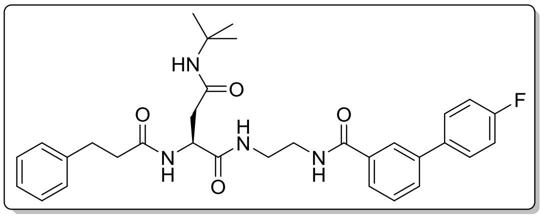

PKS21026: The title compound was synthesized by following the general procedure for HATU mediated coupling of 3-(4-fluorophenyl)benzoic acid (6.49 mg, 30 μmol) and **PKS3072** (11.9 mg, 25 μmol). After completion of reaction (1 h, temperature rose to 10 °C), mixture was purified by HPLC to give product (11.0 mg,

78%) as white solid. ^{1}H NMR (500 MHz, DMSO-*d6*) δ 8.57 (t, *J* = 5.6 Hz, 1H), 8.12 – 8.07 (m, 1H), 7.99 (d, *J* = 8.0 Hz, 1H), 7.96 (t, *J* = 5.7 Hz, 1H), 7.85 – 7.72 (m, 4H), 7.57 – 7.50 (m, 1H), 7.39 (s, 1H), 7.35 – 7.26 (m, 2H), 7.28 – 7.21 (m, 2H), 7.19 – 7.13 (m, 3H), 4.55 – 4.44 (m, 1H), 3.37 – 3.14 (m, 4H), 2.76 (t, *J* = 8.0 Hz, 2H), 2.49 – 2.35 (m, 3H), 2.32 (dd, *J* = 14.6, 7.9 Hz, 1H), 1.19 (s, 9H); ^{13}C NMR (126 MHz, DMSO-d_6) δ 171.5, 171.5, 169.0, 166.6, 162.2 (d, *J* = 244.8 Hz), 141.3, 139.2, 136.1, 135.3, 129.4, 129.2, 129.0 (d, *J* = 9.6 Hz), 128.4, 128.2, 126.5, 126.0, 125.5, 115.9 (d, *J* = 21.5 Hz), 50.4, 50.2, 39.1, 38.8, 38.7, 37.0, 31.1, 28.5; ^{19}F NMR (471 MHz, DMSO-d_6) δ -117.33 – -117.44 (m). HRMS calc. for $C_{32}H_{37}FN_4O_4Na$ [M+Na]$^+$: 583.2697. Found: 583.2701.

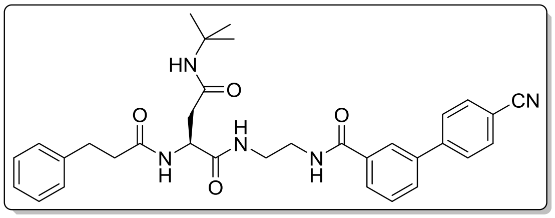

PKS21028: The title compound was synthesized by following the general procedure for HATU mediated coupling of 3-(4-cyanophenyl)benzoic acid (6.7 mg, 30 μmol) and **PKS3072** (11.9 mg, 25 μmol). After completion of reaction, mixture was purified by HPLC to give product (11.0 mg, 78%) as white solid. ^{1}H NMR (500 MHz, DMSO-*d6*) δ 8.61 (t, *J* = 5.6 Hz, 1H), 8.19 (t, *J* = 1.9 Hz, 1H), 8.01 (d, *J* = 8.0 Hz, 1H), 7.99 – 7.93 (m, 5H), 7.91 (d, *J* = 7.8 Hz, 2H), 7.59 (t, *J* = 7.8 Hz, 1H), 7.39 (s, 1H), 7.28 – 7.21 (m, 2H), 7.19 – 7.13 (m, 3H), 4.54 – 4.46 (m, 1H), 3.45 – 3.15 (m, 4H), 2.76 (t, *J* = 8.0 Hz, 2H), 2.45 (dd, *J* = 14.5, 5.9 Hz, 1H), 2.42 – 2.38 (m, 2H), 2.32 (dd, *J* = 14.5, 7.9 Hz, 1H), 1.19 (s, 9H); ^{13}C NMR (126 MHz, DMSO) δ 171.3, 171.2, 168.9, 166.1, 144.0, 141.3, 138.2, 135.4, 132.9, 129.7, 129.2, 128.3, 128.1, 127.7, 127.7, 125.8, 125.8, 118.8, 110.4, 50.2, 50.0, 38.9, 38.7, 38.6, 36.9, 31.0, 28.4. ES$^+$ calc. for $C_{33}H_{38}N_5O_4$ [M+H]$^+$: 568.3. Found: 568.6.

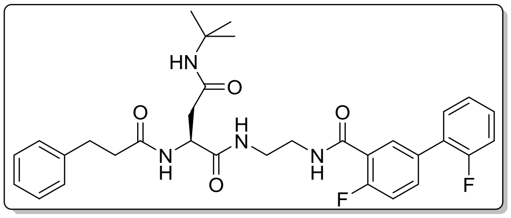

PKS21187: The title compound was synthesized by following the general procedure for HATU mediated coupling of 2-fluoro-5-(2-fluorophenyl)benzoic acid (11.7 mg, 50 μmol) and **PKS3072** (23.8 mg, 50 μmol). After completion of reaction, mixture was purified by HPLC to give product (24.8 mg, 86%) as white solid. ^{1}H NMR (500 MHz, DMSO-*d6*) δ 8.38 (t, *J* = 4.6 Hz, 1H), 7.96 (d, *J* = 8.0 Hz, 1H), 7.92 (t, *J* = 5.7 Hz, 1H), 7.82 – 7.78 (m, 1H), 7.71 – 7.66 (m, 1H), 7.58 – 7.53 (m, 1H), 7.47 – 7.41 (m, 1H), 7.41 – 7.28 (m, 4H), 7.27 – 7.22 (m, 2H), 7.19 – 7.13 (m, 3H), 4.53 – 4.42 (m, 1H), 3.38 – 3.29 (m, 2H), 3.29 – 3.23 (m, 1H), 3.22 – 3.15 (m,

1H), 2.76 (t, *J* = 7.9 Hz, 2H), 2.46 – 2.36 (m, 3H), 2.30 (dd, *J* = 14.6, 7.9 Hz, 1H), 1.18 (s, 9H); ^{13}C NMR (126 MHz, DMSO-d_6) δ 171.3, 171.2, 168.8, 163.6, 159.0 (d, *J* = 247.4 Hz), 158.8 (d, *J* = 251.0 Hz), 141.3, 132.8 – 132.5 (m), 131.4 – 131.2 (m), 130.8, 130.3, 130.0 (d, *J* = 7.9 Hz), 128.3, 128.1, 126.7, 125.8, 125.0 (d, *J* = 2.9 Hz), 124.2 (d, *J* = 14.5 Hz), 116.5 (d, *J* = 22.1 Hz), 116.1 (d, *J* = 23.1 Hz), 50.1, 50.0, 39.0, 38.6, 38.5, 36.9, 30.9, 28.4; ^{19}F NMR (471 MHz, DMSO-d_6) δ -117.8 – -117.9 (m), -120.7 – -120.9 (m). HRMS calc. for $C_{32}H_{36}F_2N_4O_4Na$ $[M+Na]^+$: 601.2602. Found: 601.2601.

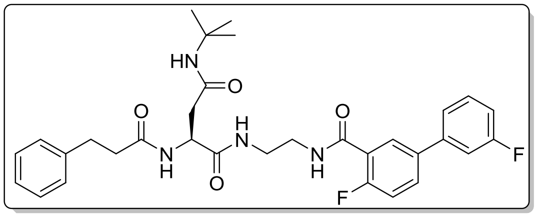

PKS21195: The title compound was synthesized by following the general procedure for HATU mediated coupling of 2-fluoro-5-(3-fluorophenyl)benzoic acid (14.3 mg, 61 μmol) and **PKS3072** (29.0 mg, 61 μmol). After completion of reaction, mixture was purified by HPLC to give product (28.5 mg, 81%) as white solid. ^1H NMR (500 MHz, DMSO-*d6*) δ 8.41 (t, *J* = 5.6 Hz, 1H), 8.00 (d, *J* = 8.0 Hz, 1H), 7.97 – 7.89 (m, 2H), 7.88 – 7.79 (m, 1H), 7.59 – 7.53 (m, 2H), 7.53 – 7.46 (m, 1H), 7.41 – 7.32 (m, 2H), 7.27 – 7.18 (m, 3H), 7.17 – 7.12 (m, 3H), 4.54 – 4.44 (m, 1H), 3.40 – 3.15 (m, 4H), 2.76 (t, *J* = 8.0 Hz, 2H), 2.45 (dd, *J* = 14.5, 6.0 Hz, 1H), 2.42 – 2.36 (m, 2H), 2.32 (dd, *J* = 14.5, 7.9 Hz, 1H), 1.18 (s, 9H); ^{13}C NMR (126 MHz, DMSO-d_6) δ 171.3, 171.2, 168.9, 163.7, 162.7 (d, *J* = 243.3 Hz), 159.1 (d, *J* = 250.7 Hz), 141.3, 141.0 (d, *J* = 7.7 Hz), 135.0, 130.9 (d, *J* = 8.0 Hz), 130.5 (d, *J* = 8.9 Hz), 128.3, 128.3, 128.1, 125.8, 124.5 (d, *J* = 14.5 Hz), 122.8, 116.8 (d, *J* = 21.9 Hz), 114.4 (d, *J* = 21.6 Hz), 113.4, 50.2, 50.0, 38.9, 38.6, 38.6, 36.9, 30.9, 28.4; ^{19}F NMR (471 MHz, DMSO-d_6) δ -114.6 – -114.8 (m), -117.8 – -118.1 (m). HRMS calc. for $C_{32}H_{36}F_2N_4O_4Na$ $[M+Na]^+$: 601.2602. Found: 601.2600

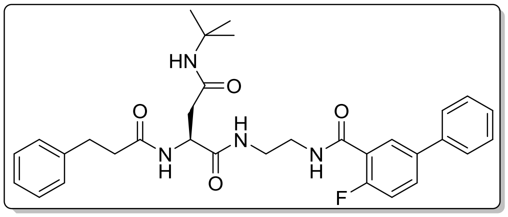

PKS21196: The title compound was synthesized by following the general procedure for HATU mediated coupling of 2-fluoro-5-phenyl-benzoic acid (13.2 mg, 61 μmol) and **PKS3072** (29.0 mg, 61 μmol). After completion of reaction, mixture was purified by HPLC to give product (30.0 mg, 88%) as white solid. ^1H NMR (500 MHz, DMSO-*d6*) δ 8.40 (t, *J* = 5.6 Hz, 1H), 8.00 (d, *J* = 8.0 Hz, 1H), 7.94 (t, *J* = 5.7 Hz, 1H), 7.90 (dd, *J* = 6.9, 2.4 Hz, 1H), 7.82 – 7.77 (m, 1H), 7.69 (d, *J* = 7.6 Hz, 2H), 7.49 – 7.44 (m, 2H), 7.40 – 7.33 (m, 3H), 7.27 – 7.21 (m, 2H), 7.18 – 7.13 (m, 3H), 4.54 – 4.45 (m, 1H), 3.39 – 3.15 (m, 4H), 2.76 (t, *J* = 8.0 Hz, 2H), 2.45 (dd, *J* = 14.5, 5.9 Hz, 1H), 2.42 – 2.37 (m, 2H), 2.32 (dd, *J* = 14.5, 7.9 Hz, 1H), 1.18 (s, 9H);

^{13}C NMR (126 MHz, DMSO-d_6) δ 171.3, 171.2, 168.9, 163.9, 158.8 (d, *J* = 250.7 Hz), 141.3, 138.5, 136.4, 130.3 (d, *J* = 8.9 Hz), 129.0, 128.3, 128.1 (d, *J* = 3.1 Hz), 128.1, 127.7, 126.7, 125.8, 124.4 (d, *J* = 14.5 Hz), 116.7 (d, *J* = 23.0 Hz), 50.2, 50.0, 38.9, 38.6, 38.6, 36.9, 30.9, 28.4; ^{19}F NMR (471 MHz, DMSO-d_6) δ -119.1 – -119.3 (m). HRMS calc. for $C_{32}H_{37}FN_4O_4Na$ $[M+Na]^+$: 583.2697. Found: 583.2697.

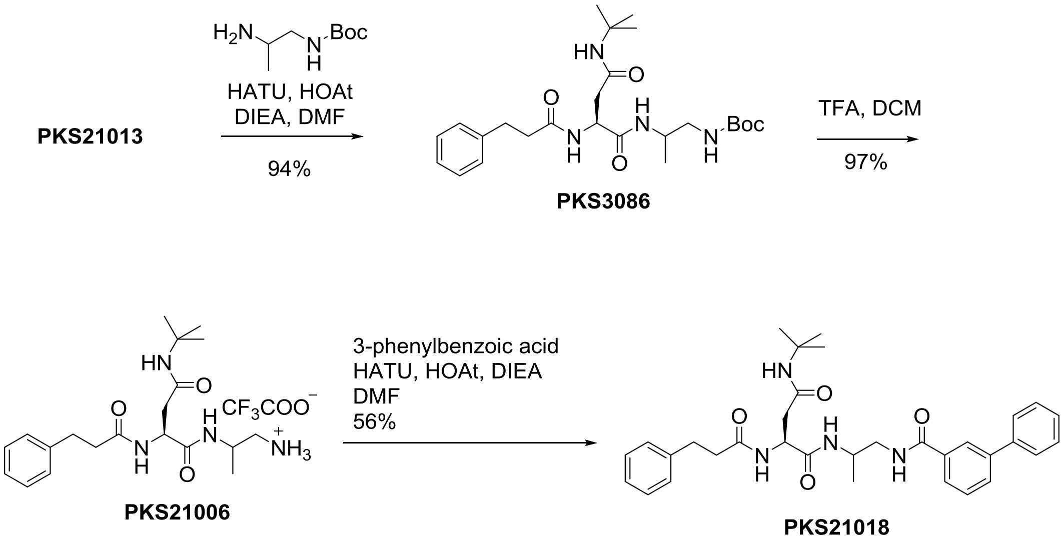

Scheme 2

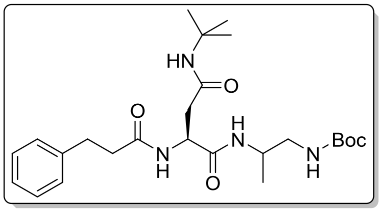

PKS3086: The title compound was synthesized by following the general procedure of HATU mediated coupling of **PKS21013** (32.0 mg, 0.1 mmol) and (±) *tert*-butyl (2-aminopropyl)carbamate (17.4 mg, 0.1 mmol). After completion of reaction, water was added. The white precipitate formed, was filtered and dried in air to give product (44.9 mg, 94%) as white solid. Product was used in next step without further purification.^{1}H NMR (500 MHz, DMSO-d_6) δ 7.91 (d, *J* = 8.0 Hz, 1H), 7.56 (d, *J* = 8.1 Hz, 1H), 7.37 (s, 1H), 7.30 – 7.23 (m, 2H), 7.22 – 7.13 (m, 3H), 6.70 (t, *J* = 6.0 Hz, 1H), 4.50 – 4.41 (m, 1H), 3.81 – 3.70 (m, 1H), 2.94 (t, *J* = 6.1 Hz, 2H), 2.79 (t, *J* = 7.9 Hz, 2H), 2.45 – 2.34 (m, 3H), 2.30 (dd, *J* = 14.7, 7.5 Hz, 1H), 1.37 (s, 9H), 1.22 (s, 9H), 0.96 (d, *J* = 6.7 Hz, 3H). ES^+ calc. for $C_{25}H_{41}N_4O_5$ $[M+H]^+$: 477.3. Found: 477.6.

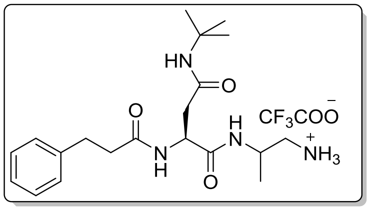

PKS21006: The title compound was synthesized by following the general procedure for Boc-deprotection of **PKS3086** (40.0 mg, 84 µmol). Isolated crude was dried under vacuum and triturated with diethyl ether to give a white solid. The diethylether was decanted and white solid was dried under vacuum to give product (40 mg, 97%) as white solid. Product was used in next step without further purification. ^{1}H NMR (500 MHz, DMSO-d_6; A mixture of diastereomers) δ 8.11 (d, *J* = 7.2 Hz, 1H), 7.86 (d, *J* = 8.4 Hz, 1H), 7.55 (s, 1H), 7.30 – 7.24 (m, 2H), 7.22 – 7.15 (m, 3H), 4.46 – 4.32 (m, 1H), 4.09 – 3.93 (m, 1H), 2.87 (dd, *J* = 13.4, 5.1 Hz, 1H), 2.84 – 2.72 (m, 3H), 2.47 – 2.39 (m, 4H), 1.23 (s, 9H), 1.11 – 1.03 (m, 3H).

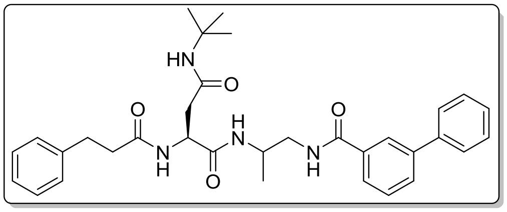

PKS21018: The title compound was synthesized by following the general procedure for HATU mediated coupling of 3-phenyl-benzoic acid (4.8 mg, 24 µmol) and **PKS21006** (9.8 mg, 20 µmol). After completion of reaction, mixture was purified by HPLC to give product (6.2 mg, 56%) as white solid. ^{1}H NMR (500 MHz, DMSO-*d6*) δ 8.51 (t, *J* = 5.9 Hz, 1H), 8.12 (t, *J* = 1.9 Hz, 1H), 7.94 (d, *J* = 7.8 Hz, 1H), 7.86 – 7.79 (m, 2H), 7.77 (d, *J* = 7.9 Hz, 1H), 7.74 – 7.69 (m, 2H), 7.53 (t, *J* = 7.7 Hz, 1H), 7.51 – 7.44 (m, 2H), 7.43 – 7.34 (m, 2H), 7.27 – 7.21 (m, 2H), 7.19 – 7.12 (m, 3H), 4.52 – 4.44 (m, 1H), 4.00 – 3.91 (m, 1H), 3.44 – 3.36 (m, 1H), 3.30 – 3.24 (m, 1H), 2.75 (t, *J* = 8.0 Hz, 2H), 2.46 – 2.35 (m, 3H), 2.32 (dd, *J* = 14.7, 7.6 Hz, 1H), 1.20 (s, 9H), 1.06 (d, *J* = 6.6 Hz, 3H); ^{13}C NMR (126 MHz, DMSO) δ 171.2, 170.6, 168.8, 166.6, 141.3, 140.2, 139.6, 135.2, 129.3, 129.0, 128.9, 128.3, 128.1, 127.7, 126.8, 126.4, 125.8, 125.4, 50.3, 50.0, 45.1, 43.9, 38.6, 36.8, 30.9, 28.4, 17.8. HRMS calc. for $C_{33}H_{40}N_4O_4Na$ $[M+Na]^+$: 579.2947. Found: 579.2958.

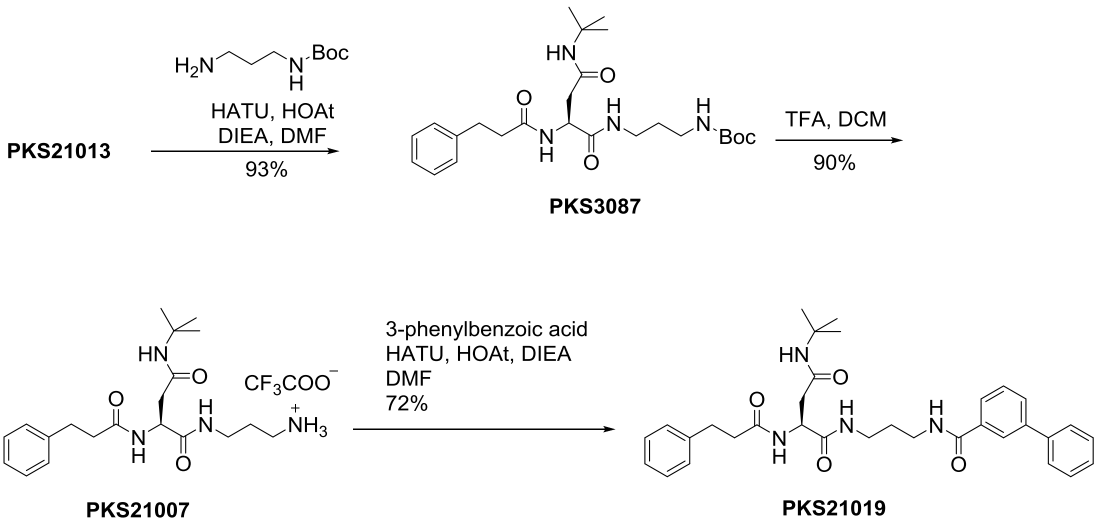

Scheme 3

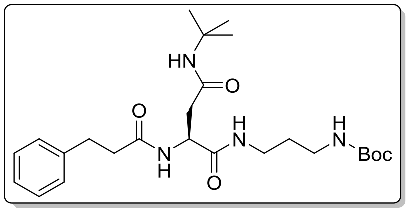

PKS3087: The title compound was synthesized by following the general procedure of HATU mediated coupling of **PKS21013** (32.0 mg, 0.1 mmol) and *tert*-butyl *N*-(3-aminopropyl)carbamate (17.4 mg, 0.1 mmol). After completion of reaction, water was added. The white precipitate formed, was filtered and dried in air to give product (44.3 mg, 93%) as white solid. Product was used in next step without further purification. ^{1}H NMR (500 MHz, DMSO-d_6) δ 7.99 – 7.94 (m, 1H), 7.69 (t, *J* = 5.9 Hz, 1H), 7.34 (s, 1H), 7.29 – 7.23 (m, 2H), 7.21 – 7.14 (m, 3H), 6.74 (t, *J* = 5.9 Hz, 1H), 4.52 – 4.42 (m, 1H), 3.03 – 2.98 (m, 2H), 2.92 – 2.86 (m, 2H), 2.82 – 2.77 (m, 2H), 2.46 – 2.36 (m, 3H), 2.28 (dd, *J* = 14.6, 8.0 Hz, 1H), 1.50 – 1.41 (m, 2H), 1.37 (s, 9H), 1.21 (s, 9H). ES$^+$ calc. for $C_{25}H_{41}N_4O_5$ $[M+H]^+$: 477.3. Found: 477.6.

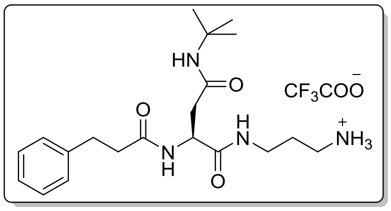

PKS21007: The title compound was synthesized by following the general procedure for Boc-deprotection of **PKS3087** (44.3 mg, 93 μmol). After completion of reaction (3h), excess trifluoroacetic acid and dichloromethane were evaporated. Crude was dried under vacuum and triturated with diethyl ether to give a white solid. The diethylether was decanted and white solid was dried under vacuum to give product (41.0 mg, 90%) as white solid. Product was used in next step without

further purification. ^{1}H NMR (500 MHz, DMSO-d_6) δ 8.04 (d, *J* = 7.8 Hz, 1H), 7.91 (t, *J* = 6.0 Hz, 1H), 7.73 (br, 3H), 7.41 (s, 1H), 7.33 – 7.22 (m, 2H), 7.23 – 7.11 (m, 3H), 4.52 – 4.40 (m, 1H), 3.15 – 3.02 (m, 2H), 2.84 – 2.68 (m, 4H), 2.46 – 2.37 (m, 3H), 2.32 (dd, *J* = 14.7, 8.0 Hz, 1H), 1.72 – 1.59 (m, 2H), 1.22 (s, 9H).

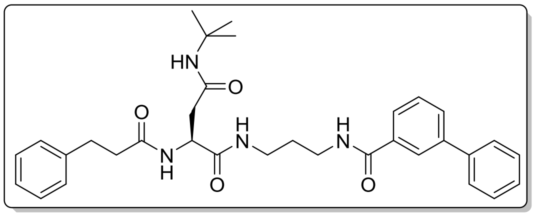

PKS21019: The title compound was synthesized by following the general procedure for HATU mediated coupling of 3-phenyl-benzoic acid (4.8 mg, 24 μmol) and **PKS21007** (9.8 mg, 20 μmol). After completion of reaction, mixture was purified by HPLC to give product (11.1 mg, 72%) as white solid. ^{1}H NMR (500 MHz, DMSO-*d6*) δ 8.57 (t, *J* = 5.8 Hz, 1H), 8.11 (t, *J* = 1.9 Hz, 1H), 8.00 (d, *J* = 8.0 Hz, 1H), 7.86 – 7.78 (m, 3H), 7.75 – 7.69 (m, 2H), 7.58 – 7.53 (m, 1H), 7.53 – 7.47 (m, 2H), 7.43 – 7.38 (m, 1H), 7.35 (s, 1H), 7.27 – 7.21 (m, 2H), 7.21 – 7.12 (m, 3H), 4.53 – 4.44 (m, 1H), 3.31 – 3.26 (m, 2H), 3.15 – 3.08 (m, 2H), 2.84 – 2.77 (m, 2H), 2.47 – 2.38 (m, 3H), 2.31 (dd, *J* = 14.6, 8.0 Hz, 1H), 1.67 – 1.61 (m, 2H), 1.21 (s, 9H); ^{13}C NMR (126 MHz, DMSO) δ 171.2, 171.0, 168.8, 166.1, 141.3, 140.2, 139.6, 135.2, 129.3, 129.0, 129.0, 128.3, 128.1, 127.6, 126.8, 126.3, 125.8, 125.3, 50.2, 50.0, 38.6, 36.9, 36.6, 36.3, 31.0, 29.2, 28.4. HRMS calc. for $C_{33}H_{40}N_4O_4Na$ $[M+Na]^+$: 579.2947. Found: 579.2953.

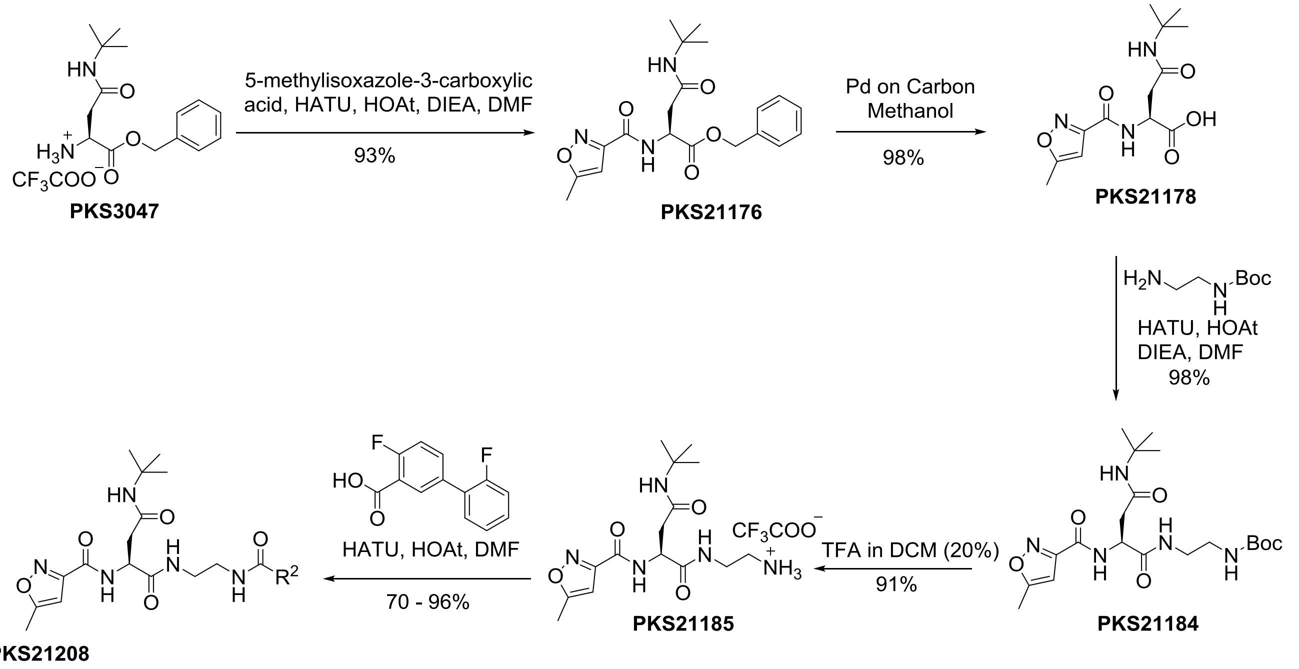

Scheme 4

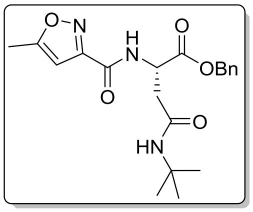

PKS21176: The title compound was synthesized by following the general procedure for HATU mediated coupling of 5-methylisoxazole-3-carboxylic acid (139.8 mg, 1.10 mmol) and **PKS3047** (431.0 mg, 1.10 mmol). After completion of reaction, water was added. The white precipitate formed, was filtered, washed with water and dried in air to give product (395 mg, 93%) as white solid. Product was pure (by NMR) and used in next step without further purification. ^{1}H NMR (500 MHz, Chloroform-*d*) δ 8.00 (d, *J* = 8.3 Hz, 1H), 7.37 – 7.26 (m, 5H), 6.40 (d, *J* = 1.1 Hz, 1H), 5.30 (br, 1H), 5.25 (d, *J* = 12.4 Hz, 1H), 5.19 (d, *J* = 12.4 Hz, 1H), 4.97 (dt, *J* = 8.7, 4.5 Hz, 1H), 2.89 (dd, *J* = 15.6, 4.6 Hz, 1H), 2.71 (dd, *J* = 15.6, 4.5 Hz, 1H), 2.47 (s, 3H), 1.28 (s, 9H). ES^{+} calc. for $C_{20}H_{26}N_3O_5$ $[M+H]^+$: 388.2. Found: 388.1

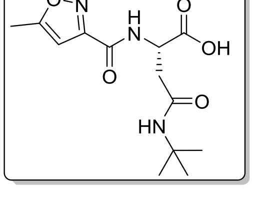

PKS21178: The title compound was synthesized by following the general procedure for *O*-debenzylation of **PKS21176** (195.0 mg, 0.503 mmol). The product (146 mg, 98%) was isolated as colorless gum and used in next step without further purification. ^{1}H NMR (500 MHz, Chloroform-*d*) δ 8.04 (d, *J* = 6.5 Hz, 1H),

6.46 (s, 1H), 6.37 (s, 1H), 4.83 – 4.75 (m, 1H), 2.93 (dd, J = 15.6, 3.6 Hz, 1H), 2.78 (dd, J = 15.6, 8.1 Hz, 1H), 2.45 (s, 3H), 1.32 (s, 9H). ES^+ calc. for $C_{13}H_{20}N_3O_5$ $[M+H]^+$: 298.1. Found: 298.0

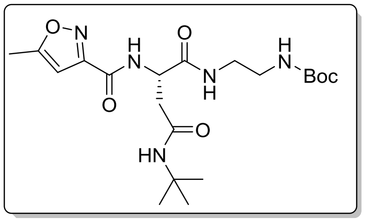

PKS21184: The title compound was synthesized by following the general procedure of HATU mediated coupling of **PKS21178** (145.0 mg, 0.488 mmol) and *N*-boc ethylenediamine (78.1 mg, 0.488 mmol). After completion of reaction, water was added. Mixture was extracted with ethyl acetate twice. Combined organic layer was washed with aq. $NaHCO_3$, water, 1N HCl, saturated brine, dried over anhydrous sodium sulfate and evaporated to give product (210.0 mg, 98%) as off-white solid. Product was used in next step without further purification. ^1H NMR (500 MHz, Chloroform-*d*) δ 8.32 (d, J = 7.7 Hz, 1H), 7.44 (br, 1H), 6.41 (s, 1H), 6.05 – 5.82 (m, 1H), 5.13 (br, 1H), 4.90 – 4.80 (m, 1H), 3.41 – 3.29 (m, 2H), 3.28 – 3.18 (m, 2H), 2.86 – 2.76 (m, 1H), 2.65 – 2.56 (m, 1H), 2.46 (s, 3H), 1.39 (s, 9H), 1.32 (s, 9H). ES^+ calc. for $C_{20}H_{34}N_5O_6$ $[M+H]^+$: 440.3. Found: 440.2

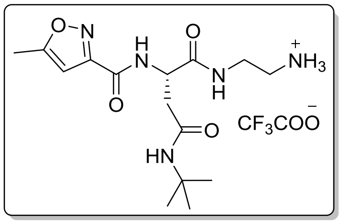

PKS21185: The title compound was synthesized by following the general procedure for Boc-deprotection of **PKS21184** (210.0 mg, 0.478 mmol). Isolated crude was dried under vacuum and triturated with diethyl ether to give a white solid. The diethylether was decanted and white solid was dried under vacuum to give product (197.0 mg, 91%) as white solid. Product was used in next step without further purification. ^1H NMR (500 MHz, DMSO-*d6*) δ 8.54 (d, J = 8.1 Hz, 1H), 8.25 – 8.16 (m, 1H), 7.78 (br, 3H), 7.60 (s, 1H), 6.56 (d, J = 1.0 Hz, 1H), 4.73 – 4.62 (m, 1H), 3.35 – 3.23 (m, 2H), 2.89 – 2.81 (m, 2H), 2.59 (dd, J = 13.3, 5.9 Hz, 1H), 2.55 (dd, J = 13.3, 4.8 Hz, 1H), 2.47 (s, 3H), 1.20 (s, 9H). ES^+ calc. for $C_{15}H_{26}N_5O_4$ $[M+H]^+$: 340.2. Found: 340.1

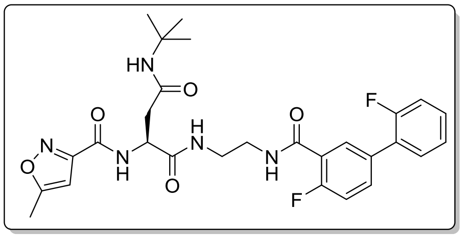

PKS21208: The title compound was synthesized by following the general procedure for HATU mediated coupling of 2-fluoro-5-(2-fluorophenyl)benzoic acid (11.7 mg, 50 μmol) and **PKS21185** (22.7 mg, 50 μmol). After completion of reaction, mixture was purified by preparative LCMS to give product (21.0 mg, 76%) as

white solid. ^{1}H NMR (500 MHz, DMSO-*d6*) δ 8.52 (d, *J* = 8.0 Hz, 1H), 8.38 (d, *J* = 5.9 Hz, 1H), 8.16 (t, *J* = 5.6 Hz, 1H), 7.78 (d, *J* = 6.7 Hz, 1H), 7.72 – 7.67 (m, 1H), 7.60 – 7.54 (m, 1H), 7.49 (s, 1H), 7.47 – 7.42 (m, 1H), 7.42 – 7.36 (m, 1H), 7.36 – 7.28 (m, 2H), 6.49 (s, 1H), 4.69 – 4.61 (m, 1H), 3.43 – 3.15 (m, 4H), 2.56 (dd, *J* = 14.4, 8.2 Hz, 1H), 2.52 – 2.46 (m, 1H), 2.44 (s, 3H), 1.17 (s, 9H). ^{13}C NMR (126 MHz, DMSO-d_6) δ 171.2, 170.4, 168.9, 163.5, 159.0 (d, *J* = 247.1 Hz), 158.8 (d, *J* = 251.1 Hz), 158.6, 158.3, 132.8 – 132.5 (m), 131.3, 130.8 (d, *J* = 3.3 Hz), 130.4, 130.0 (d, *J* = 7.4 Hz), 126.6 (d, *J* = 12.7 Hz), 125.0 (d, *J* = 2.7 Hz), 124.1 (d, *J* = 14.6 Hz), 116.5 (d, *J* = 23.0 Hz), 116.1 (d, *J* = 21.8 Hz), 101.3, 50.5, 50.1, 39.0, 38.5, 38.1, 28.3, 11.8. ^{19}F NMR (471 MHz, DMSO-d_6) δ -117.69 – -117.85 (m), -120.75 – -120.88 (m). HRMS calc. for $C_{28}H_{31}F_2N_5O_5Na$ $[M+Na]^+$: 578.2191. Found: 578.2177.

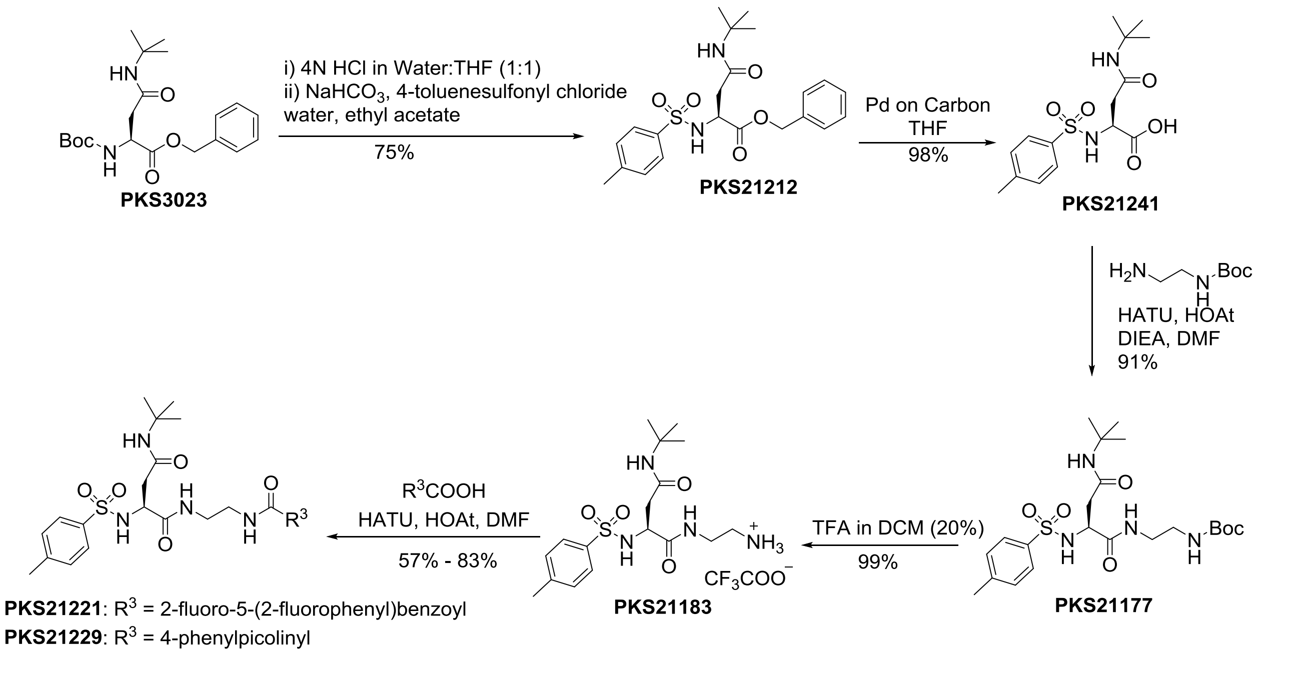

Scheme 5

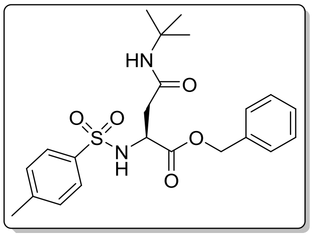

PKS21212: **PKS3023** (1.60 g, 4.23 mmol) was dissolved in water: tetrahydrofuran (1:1, 20 mL) mixture and 5 mL of HCl (12 *N*) was added. The mixture was stirred at room temperature for 4 hours. Tetrahydrofuran was evaporated and the resulting solution was diluted with 10 mL water and basified with pinch-wise addition of solid sodium bicarbonate (approx. 12 g).

4-Toluenesulfonyl chloride (1.61 g, 8.46 mmol) and 50 mL ethyl acetate were added. The biphasic mixture was vigorously stirred at room temperature for 2 hours. The layers were separated and aqueous layer was washed with ethyl acetate. Combined ethyl acetate layer was evaporated and purified by combi-flash to give product (1.37 g, 75%) as white solid. ^{1}H NMR (500 MHz, Chloroform-*d*) δ 7.75 – 7.68 (m, 2H), 7.35 – 7.28 (m, 3H), 7.24 – 7.17 (m, 4H), 5.90 (d, *J* = 7.8 Hz, 1H), 5.31 (br, 1H), 5.04 (d, *J* = 12.2 Hz, 1H), 5.00 (d, *J* = 12.2 Hz, 1H), 4.12 – 4.04 (m, 1H), 2.80 (dd, *J* = 15.3, 4.1 Hz, 1H), 2.62 (dd, *J* = 15.3, 4.6 Hz, 1H), 2.39 (s, 3H), 1.29 (s, 9H). ES$^+$ calc. for $C_{22}H_{29}N_2O_5S$ [M+H]$^+$: 433.2. Found: 433.2

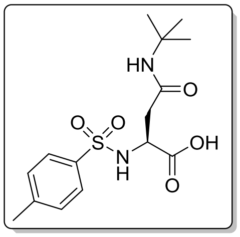

PKS21241: The title compound was synthesized by following the general procedure for *O*-debenzylation of **PKS21212** (1.37 g, 3.17 mmol) in tetrahydrofuran (15.00 mL). The product (1.06 g, 98%) was isolated as white solid and used in next step without further purification. ^{1}H NMR (500 MHz, DMSO-d_6) δ 12.55 (s, 1H), 7.86 (d, *J* = 8.7 Hz, 1H), 7.66 (d, *J* = 7.8 Hz, 2H), 7.41 (s, 1H), 7.33 (d, *J* = 7.8 Hz, 2H), 4.09 – 4.02 (m, 1H), 2.42 (dd, *J* = 15.1, 6.8 Hz, 1H), 2.36 (s, 3H), 2.24 (dd, *J* = 15.1, 6.5 Hz, 1H), 1.17 (s, 9H). ES$^+$ calc. for $C_{15}H_{23}N_2O_5S$ [M+H]$^+$: 343.1. Found: 343.2

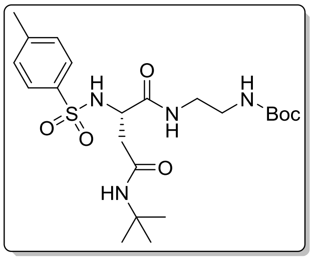

PKS21177: The title compound was synthesized by following the general procedure of HATU mediated coupling of **PKS21241** (342.4 mg, 1.00 mmol) and *N*-boc-ethylenediamine (176.2 mg, 1.10 mmol). After completion of reaction, water was added and mixture was srirred for 30 minutes at room temperature. The white precipitate formed, was filtered, washed with water and dried in air to give product (441.0 mg, 91%) as white solid. Product was used in next step without further purification. ^{1}H NMR (500 MHz, Chloroform-*d*) δ 7.76 (d, *J* = 8.4 Hz, 2H), 7.34 – 7.30 (m, 3H), 6.71 (br, 1H), 5.51 (br, 1H), 4.98 (br, 1H), 3.93 – 3.84 (m, 1H), 3.34 – 3.26 (m, 2H), 3.23 – 3.15 (m, 2H), 2.67 (dd, *J* = 15.1, 4.2 Hz, 1H), 2.43 (s, 3H), 2.16 – 2.04 (m, 1H), 1.45 (s, 9H), 1.27 (s, 9H). ES$^+$ calc. for $C_{22}H_{37}N_4O_6S$ [M+H]$^+$: 485.2. Found: 485.3

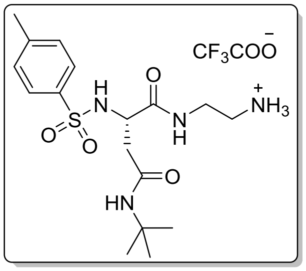

PKS21183: The title compound was synthesized by following the general procedure for Boc-deprotection of **PKS21177** (431.0 mg, 0.889 mmol). Isolated crude was dried under vacuum and triturated with diethyl ether to give a white solid. The diethylether was decanted and white solid was dried under vacuum to give product (440.0 mg, 99%) as white solid. Product was used in next step without further purification. ^{1}H NMR (500 MHz, DMSO-*d6*) δ 8.07 (t, *J* = 5.9 Hz, 1H), 7.88 (d, *J* = 8.4 Hz, 1H), 7.69 (br, 3H), 7.66 (d, *J* = 8.0 Hz, 2H), 7.57 (s, 1H), 7.34 (d, *J* = 8.0 Hz, 2H), 3.96 – 3.87 (m, 1H), 3.21 – 3.11 (m, 1H), 3.10 – 3.00 (m, 1H), 2.80 – 2.64 (m, 2H), 2.37 (s, 3H), 2.34 (dd, *J* = 14.8, 7.9 Hz, 1H), 2.27 (dd, *J* = 14.8, 6.1 Hz, 1H), 1.18 (s, 9H). ES^{+} calc. for $C_{17}H_{29}N_{4}O_{4}S$ $[M+H]^{+}$: 385.2. Found: 385.3

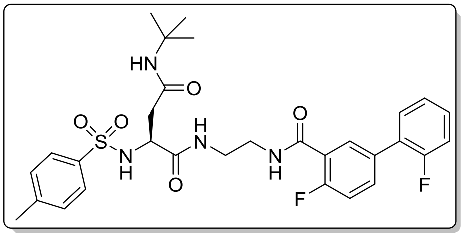

PKS21221: The title compound was synthesized by following the general procedure for HATU mediated coupling of 2-fluoro-5-(2-fluorophenyl)benzoic acid (11.7 mg, 50 mmol) and **PKS21183** (24.9 mg, 50 mmol). After completion of reaction, mixture was purified by preparative LCMS to give product (25.0 mg, 83%) as white solid. 1H NMR (500 MHz, DMSO-*d6*) δ 8.27 (t, *J* = 5.7 Hz, 1H), 7.95 (t, *J* = 5.8 Hz, 1H), 7.80 – 7.74 (m, 2H), 7.72 – 7.67 (m, 1H), 7.64 (d, *J* = 7.5 Hz, 2H), 7.59 – 7.53 (m, 1H), 7.48 – 7.28 (m, 7H), 4.02 – 3.91 (m, 1H), 3.24 – 3.11 (m, 2H), 3.09 – 2.93 (m, 2H), 2.35 (s, 3H), 2.31 (dd, *J* = 14.7, 7.0 Hz, 1H), 2.20 (dd, *J* = 14.7, 6.9 Hz, 1H), 1.14 (s, 9H). ^{13}C NMR (126 MHz, DMSO) δ 170.1, 168.1, 163.4, 159.0 (d, *J* = 247.2 Hz), 158.8 (d, *J* = 251.2 Hz), 142.5, 138.1, 132.8 – 132.6 (m), 131.3 (d, *J* = 2.5 Hz), 130.8, 130.3, 130.0 (d, *J* = 7.5 Hz), 129.2, 126.6, 126.6, 125.1 (d, *J* = 2.8 Hz), 124.1 (d, *J* = 14.7 Hz), 116.5 (d, *J* = 23.5 Hz), 116.2 (d, *J* = 23.1 Hz), 53.7, 50.1, 39.4, 38.8, 38.3, 28.3, 20.9. ^{19}F NMR (471 MHz, DMSO-d_6) -117.75 – -117.89 (m), -120.79 – -120.9 (m). HRMS calc. for $C_{30}H_{34}F_{2}N_{4}O_{5}SNa$ [M+Na]+: 623.2116. Found: 623.2107.

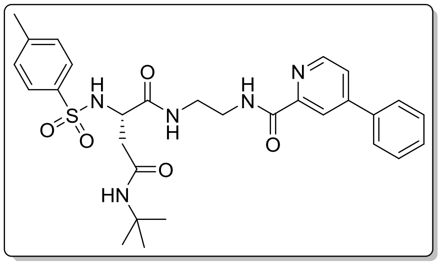

PKS21229: The title compound was synthesized by following the general procedure for HATU mediated coupling of 4-phenylpicolinic acid (10.0 mg, 50 µmol) and **PKS21183** (24.9 mg, 50 µmol). After completion of reaction, mixture was purified by preparative LCMS to give product (16.0 mg, 57%) as white solid. ^{1}H NMR (500 MHz, DMSO-*d6*) δ 8.91 (t, *J* = 6.0 Hz, 1H), 8.73 (d, *J* = 5.0 Hz, 1H), 8.31 (s, 1H), 8.03 (t, *J* = 5.7 Hz, 1H), 7.96 (dd, *J* = 4.9, 2.3 Hz, 1H), 7.88 (d, *J* = 7.4 Hz, 2H), 7.79 (br, 1H), 7.67 (d, *J* = 7.8 Hz, 2H), 7.62 – 7.50 (m, 3H), 7.38 (s, 1H), 7.30 (d, *J* = 7.8 Hz, 2H), 4.06 – 3.95 (m, 1H), 3.34 – 3.22 (m, 2H), 3.14 – 2.99 (m, 2H), 2.35 (s, 3H), 2.34 – 2.30 (m, 1H), 2.22 (dd, *J* = 14.6, 7.1 Hz, 1H), 1.18 (s, 9H); ^{13}C NMR (126 MHz, DMSO) δ 170.1, 168.0, 164.0, 150.8, 149.1, 148.6, 142.4, 138.1, 136.7, 129.7, 129.4, 129.2, 126.9, 126.6, 123.7, 119.0, 53.7, 50.0, 39.5, 38.6, 38.4, 28.4, 20.9. HRMS calc. for $C_{29}H_{35}N_5O_5SNa$ $[M+Na]^+$: 588.2257. Found: 588.2238.

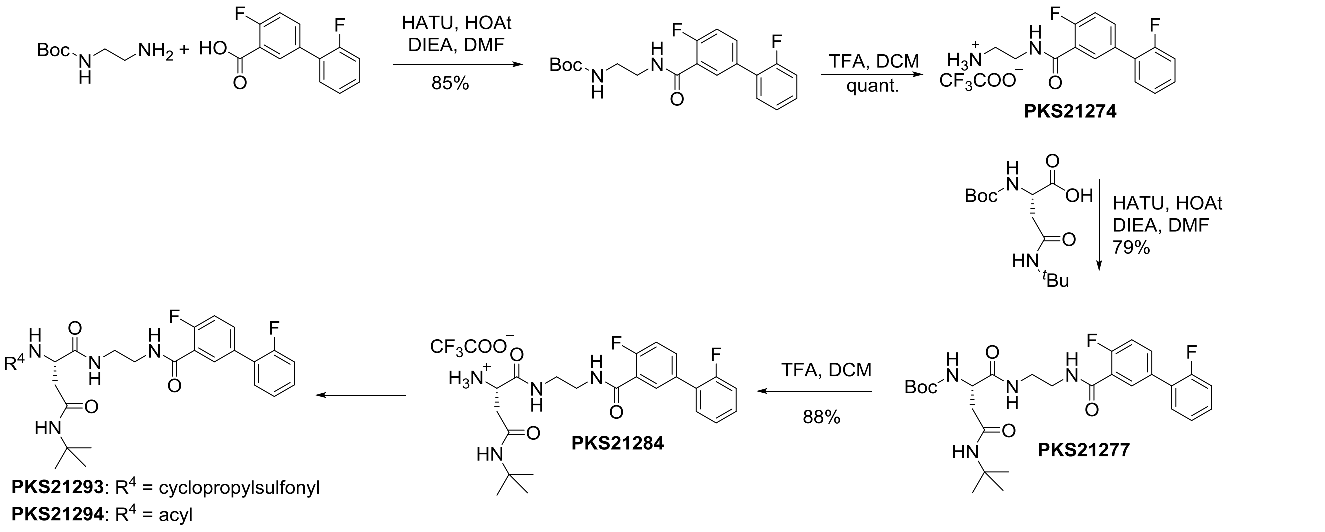

Scheme 6

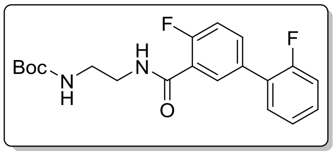

PKS21270: The title compound was synthesized by following the general procedure for HATU mediated coupling of 2-fluoro-5-(2-fluorophenyl)benzoic acid (117.1 mg, 500 µmol) and *tert*-butyl *N*-(2-aminoethyl)carbamate (88.1 mg, 550 µmol). After completion of reaction, water was added and mixture was stirred at room temperature for 30 minutes. The white precipitate appeared was filtered, washed with water and dried in air to give product (160.0 mg, 85%) as white solid. ^{1}H NMR (500 MHz, Chloroform-*d*) δ 8.22 (d, *J* = 7.3 Hz, 1H), 7.69 – 7.62 (m, 1H), 7.48 – 7.40 (m, 1H), 7.36 – 7.29 (m, 1H),

7.25 – 7.10 (m, 4H), 4.96 (br, 1H), 3.64 – 3.58 (m, 2H), 3.43 – 3.37 (m, 2H), 1.42 (s, 9H). ES$^+$ calc. for $C_{20}H_{23}F_2N_2O_3$ [M+H]$^+$: 377.2. Found 377.2.

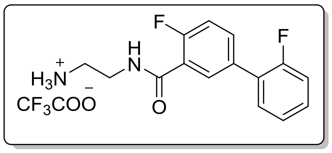

PKS21274: The title compound was synthesized by following the general procedure for Boc-deprotection of **PKS21270** (150.0 mg, 399 μmol). Isolated crude was dried under vacuum and triturated with diethyl ether to give a white solid. Diethyl ether was decanted and white solid was dried under vacuum to give product (155 mg, quant.). Product was used in next step without further purification. ^{1}H NMR (500 MHz, DMSO-*d6*) δ 8.61 – 8.50 (m, 1H), 8.04 – 7.76 (m, 4H), 7.76 – 7.67 (m, 1H), 7.61 – 7.51 (m, 1H), 7.49 – 7.39 (m, 2H), 7.38 – 7.30 (m, 2H), 3.59 – 3.46 (m, 2H), 3.07 – 2.93 (m, 2H). ES$^+$ calc. for $C_{15}H_{15}F_2N_2O$ [M+H]$^+$: 277.2. Found 277.2.

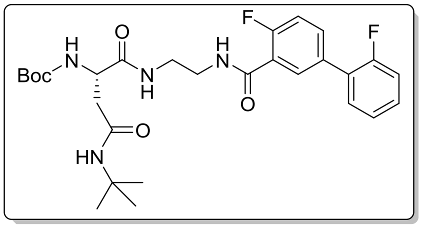

PKS21277: The title compound was synthesized by following the general procedure for HATU mediated coupling of (2*S*)-2-(*tert*-butoxycarbonylamino)-4-(*tert*-butylamino)-4-oxo-butanoic acid (28.8 mg, 100 μmol) and **PKS21274** (39.0 mg, 100 μmol). After completion of reaction, mixture was purified by preparative LCMS to give product (43.0 mg. 79%) as white solid. ^{1}H NMR (500 MHz, DMSO-*d6*) δ 8.44 – 8.33 (m, 1H), 7.98 (t, *J* = 5.6 Hz, 1H), 7.83 – 7.75 (m, 1H), 7.74 – 7.67 (m, 1H), 7.60 – 7.52 (m, 1H), 7.48 – 7.42 (m, 1H), 7.42 – 7.29 (m, 4H), 6.74 (d, *J* = 8.2 Hz, 1H), 4.24 – 4.13 (m, 1H), 3.40 – 3.24 (m, 3H), 3.24 – 3.15 (m, 1H), 2.38 (dd, *J* = 14.3, 5.4 Hz, 1H), 2.30 (dd, *J* = 14.3, 8.4 Hz, 1H), 1.34 (s, 9H), 1.19 (s, 9H). HRMS calc. for $C_{28}H_{36}F_2N_4O_5Na$ [M+Na]$^+$: 569.2551. Found: 569.2564.

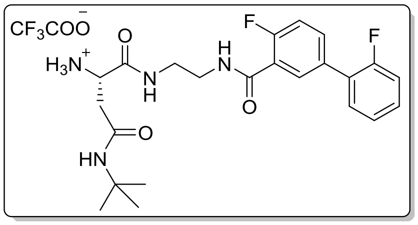

PKS21284: The title compound was synthesized by following the general procedure for Boc-deprotection of **PKS21277** (21.0 mg, 38 μmol). Isolated crude was purified by preparative LCMS to give product (19.0 mg, 88%) as colorless gum. ^{1}H NMR (500 MHz, DMSO-*d6*) δ 8.49 (t, *J* = 5.2 Hz, 1H), 8.46 – 8.41 (m, 1H), 8.10 (d, *J* = 4.8 Hz, 3H), 7.82 – 7.78 (m, 2H), 7.73 – 7.69 (m, 1H), 7.59 – 7.54 (m, 1H), 7.49 – 7.39 (m, 2H), 7.36 – 7.30 (m, 2H), 4.00 – 3.94 (m,

1H), 3.43 – 3.29 (m, 3H), 3.29 – 3.19 (m, 1H), 2.65 (dd, *J* = 16.5, 5.1 Hz, 1H), 2.55 (dd, *J* = 16.5, 7.8 Hz, 1H), 1.22 (s, 9H). ES^+ calc. for $C_{23}H_{29}F_2N_4O_3$ $[M+H]^+$: 447.2. Found: 447.3

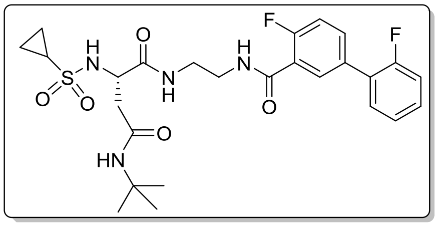

PKS21293: The title compound was synthesized by following the general procedure for *N*-sulfonamide formation of **PKS21284** (10.7 mg, 19 μmol). After completion of reaction, dichloromethane was evaporated and crude was purified by preperative LCMS to give product (9.2 mg, 88%) as white solid. ^{1}H NMR (500 MHz, DMSO-d_6) δ 8.40 – 8.35 (m, 1H), 8.12 (t, *J* = 5.6 Hz, 1H), 7.80 – 7.76 (m, 1H), 7.72 – 7.67 (m, 1H), 7.57 (td, *J* = 7.9, 1.7 Hz, 1H), 7.49 – 7.37 (m, 3H), 7.36 – 7.29 (m, 3H), 4.14 – 4.03 (m, 1H), 3.42 – 3.25 (m, 3H), 3.25 – 3.16 (m, 1H), 2.49 – 2.42 (m, 2H), 2.37 (dd, *J* = 14.8, 7.4 Hz, 1H), 1.19 (s, 9H), 0.89 – 0.78 (m, 4H). ^{13}C NMR (126 MHz, DMSO-d_6) δ 171.2, 168.5, 163.5, 159.0 (d, *J* = 247.3 Hz), 158.8 (d, *J* = 251.1 Hz), 132.9 – 132.6 (m), 131.3 (d, *J* = 2.6 Hz), 130.8, 130.3, 130.0 (d, *J* = 7.4 Hz), 126.6 (d, *J* = 12.8 Hz), 125.1 (d, *J* = 2.9 Hz), 124.2 (d, *J* = 14.6 Hz), 116.5 (d, *J* = 22.9 Hz), 116.2 (d, *J* = 22.1 Hz), 53.8, 50.1, 39.8, 39.0, 38.5, 30.2, 28.4, 5.04, 4.85. ^{19}F NMR (471 MHz, DMSO-d_6) δ -117.8 – -117.9 (m), -120.8 – -120.9 (m). HRMS calc. for $C_{26}H_{33}F_2N_4O_5S$ $[M+23]^+$: 573.1959. Found: 573.1960.

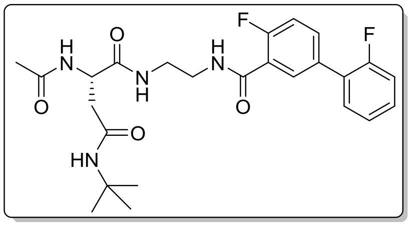

PKS21294: Hunig's base (8.7 mg, 67 μmol, 11.7 uL) and *N*,*N*-dimethylpyridin-4-amine (1.4 mg, 11 μmol) were added to a solution of **PKS21284** (12.5 mg, 22 μmol) in dichloromethane (1.00 mL) at 0 °C. The solution was stirred for 5 minutes and acetic anhydride (2.7 mg, 26.8 μmol, 2.5 uL) was added. The reaction mixture was stirred at 0 °C for 1 hour. After completion of reaction dichloromethane was evaporated and crude was purified by preparative LCMS to give product (7.1 mg, 65%) as white solid. ^{1}H NMR (500 MHz, DMSO-d_6) δ 8.43 – 8.33 (m, 1H), 7.98 (t, *J* = 5.7 Hz, 1H), 7.95 (d, *J* = 8.1 Hz, 1H), 7.81 – 7.77 (m, 1H), 7.72 – 7.67 (m, 1H), 7.57 (td, *J* = 7.9, 1.7 Hz, 1H), 7.48 – 7.42 (m, 1H), 7.39 (dd, *J* = 10.2, 8.6 Hz, 1H), 7.36 – 7.30 (m, 3H), 4.50 – 4.39 (m, 1H), 3.41 – 3.28 (m, 2H), 3.28 – 3.12 (m, 2H), 2.42 (dd, *J* = 14.5, 5.8 Hz, 1H), 2.29 (dd, *J* = 14.5, 8.1 Hz, 1H), 1.80 (s, 3H), 1.18 (s, 9H); ^{13}C NMR (126 MHz, DMSO-d_6) δ 171.4, 169.0, 168.9, 163.6, 159.0 (d, *J* = 246.9 Hz), 158.8 (d, *J* = 252.6 Hz), 132.8 – 132.5 (m), 131.4 – 131.2 (m), 130.8, 130.4, 130.0 (d, *J* = 7.5 Hz), 126.7 (d, *J* = 12.8 Hz), 125.1 (d, *J* = 2.5 Hz), 124.2 (d, *J* = 14.5 Hz), 116.5 (d, *J* = 22.1 Hz), 116.2 (d, *J* = 21.9 Hz), 50.2, 50.0, 39.0, 38.7, 38.5, 28.4, 22.6; ^{19}F NMR

(471 MHz, DMSO-d_6) δ -117.8 – -117.9 (m), -120.8 – -120.9 (m). HRMS calc. for $C_{25}H_{31}F_2N_4O_4$ $[M+23]^+$: 511.2133. Found: 511.2133.

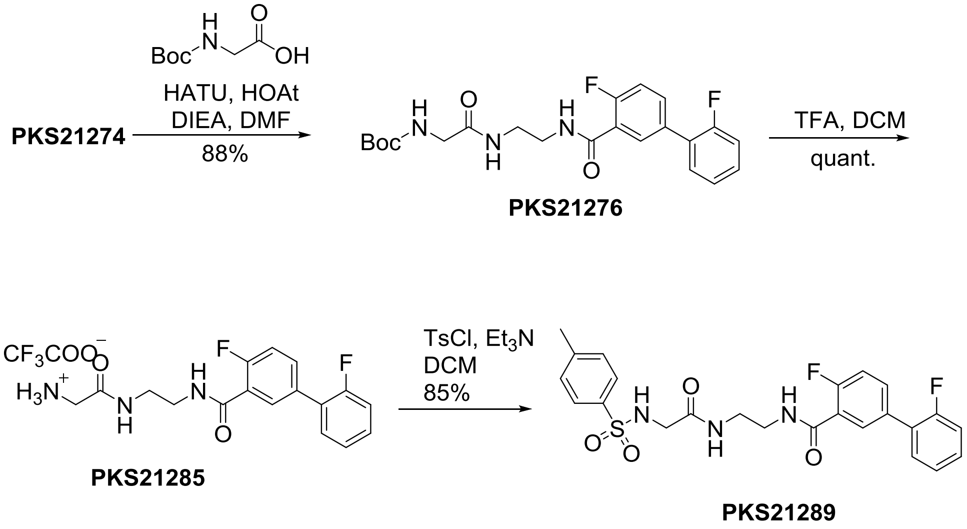

Scheme 7

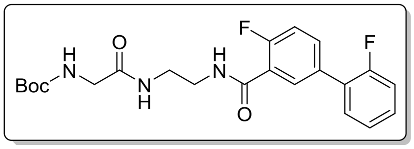

PKS21276: The title compound was synthesized by following the general procedure for HATU mediated coupling of Boc-glycine (19.3 mg, 110 μmol) and **PKS21274** (39.0 mg, 100 μmol). After completion of reaction, mixture was purified by preparative LCMS to give product (38.0 mg, 88%) as colorless solid. ^1H NMR (500 MHz, Chloroform-*d*) δ 8.23 – 8.16 (m, 1H), 7.69 – 7.62 (m, 1H), 7.47 – 7.40 (m, 1H), 7.37 – 7.31 (m, 1H), 7.26 – 7.11 (m, 4H), 6.89 (t, J = 5.7 Hz, 1H), 5.16 (br, 1H), 3.80 (d, J = 4.2 Hz, 2H), 3.67 – 3.61 (m, 2H), 3.57 – 3.51 (m, 2H), 1.41 (s, 9H). HRMS calc. for $C_{22}H_{26}F_2N_3O_4$ $[M+H]^+$: 434.1891. Found 434.1874.

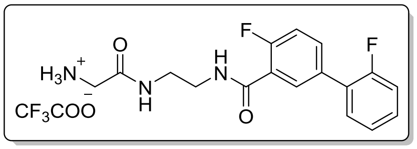

PKS21285: The title compound was synthesized by following the general procedure for Boc-deprotection of **PKS21276** (32.0 mg, 74 μmol). Isolated crude was dried under vacuum to give product (33 mg, quant.). Product was used in next step without further purification. ^1H NMR (500 MHz, DMSO-*d6*) δ 8.52 – 8.43 (m, 2H), 8.09 – 7.98 (m, 3H), 7.82 – 7.77 (m, 1H), 7.74 – 7.68 (m, 1H), 7.57 (td, J = 7.9, 1.7 Hz, 1H), 7.48 – 7.38 (m, 2H), 7.37 – 7.30 (m, 2H), 3.56 – 3.49 (m, 2H), 3.41 – 3.34 (m, 2H), 3.34 – 3.28 (m, 2H). ES^+ calc. for $C_{17}H_{18}F_2N_3O_2$ $[M+H]^+$: 334.1. Found 334.2.

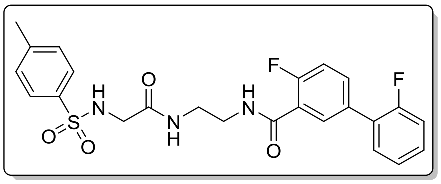

PKS21289: The title compound was synthesized by following the general procedure for sulfonamide preparation of **PKS21285** (16.00 mg, 35.77 μmol) with 4-methylbenzenesulfonyl chloride (13.6 mg, 72 μmol). After completion of reaction, dichloromethane was evaporated and crude was purified by preparative LCMS to give product (14.8 mg, 85%) as white solid. ^{1}H NMR (500 MHz, DMSO-d_6) δ 8.43 – 8.34 (m, 1H), 8.02 (t, *J* = 5.8 Hz, 1H), 7.86 (s, 1H), 7.80 – 7.74 (m, 1H), 7.73 – 7.69 (m, 1H), 7.67 (d, *J* = 8.3 Hz, 2H), 7.57 (td, *J* = 7.9, 1.9 Hz, 1H), 7.49 – 7.41 (m, 1H), 7.44 – 7.28 (m, 5H), 3.43 – 3.31 (m, 2H), 3.31 – 3.23 (m, 2H), 3.23 – 3.15 (m, 2H), 2.37 (s, 3H). ^{13}C NMR (126 MHz, DMSO-d_6) δ 167.8, 163.5, 159.0 (d, *J* = 246.9 Hz), 158.8 (d, *J* = 251.2 Hz), 142.8, 137.1, 132.7 (d, *J* = 8.7 Hz), 131.3, 130.8, 130.3, 130.0 (d, *J* = 8.5 Hz), 129.5, 126.7, 126.6 – 126.5 (m), 125.1, 124.2 (d, *J* = 14.6 Hz), 116.5 (d, *J* = 22.4 Hz), 116.2 (d, *J* = 22.1 Hz), 45.3, 39.0, 38.2, 21.0; ^{19}F NMR (471 MHz, DMSO-d_6) δ -117.8 – -118.0 (m), -120.8 – -121.0 (m). HRMS calc. for $C_{24}H_{24}F_2N_3O_4S$ $[M+23]^+$: 510.1275. Found: 510.1265.

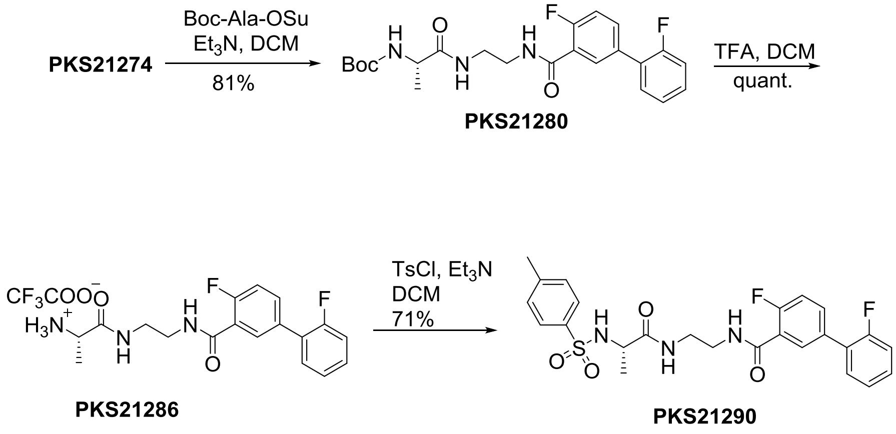

Scheme 8

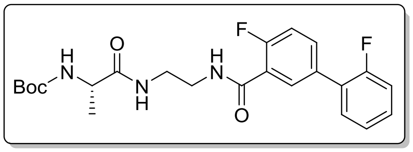

PKS21280: Triethylamine (75.8 mg, 749 μmol, 104 uL) was added to a solution of **PKS21274** (39.0 mg, 100 μmol) in dichloromethane (3.00 mL) at 0 °C. The mixture was stirred for 10 minutes and Boc-

Ala-OSu (31.5 mg, 110 μmol) was added. The reaction mixture was allowed to warm to room temperature slowly. After completion of reaction (2 hr, temperature rose to 20 °C), dichloromethane was evaporated and crude was purified by preparative LCMS to give product (36.3 mg, 81%) as white solid. ^{1}H NMR (500 MHz, DMSO-*d6*) δ 8.36 (t, *J* = 5.5 Hz, 1H), 7.92 (t, *J* = 5.5 Hz, 1H), 7.78 (dd, *J* = 7.2, 2.4 Hz, 1H), 7.73 – 7.66 (m, 1H), 7.59 – 7.53 (m, 1H), 7.48 – 7.42 (m, 1H), 7.40 (dd, *J* = 10.3, 8.5 Hz, 1H), 7.36 – 7.30 (m, 2H), 6.84 (d, *J* = 7.4 Hz, 1H), 3.96 – 3.74 (m, 1H), 3.46 – 3.13 (m, 4H), 1.34 (s, 9H), 1.15 (d, *J* = 7.2 Hz, 3H). ES$^+$ calc. for $C_{23}H_{28}F_2N_3O_4$ [M+H]$^+$: 448.2. Found 448.3.

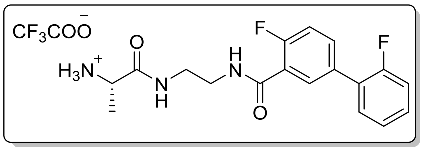

PKS21286: The title compound was synthesized by following the general procedure for Boc-deprotection of **PKS21280** (30.0 mg, 67 μmol). Ioslated crude was dried under vacuum to give product (31.0 mg, quant.). Product was used in next step without further purification. ^{1}H NMR (500 MHz, DMSO-*d6*) δ 8.50 (t, *J* = 5.9 Hz, 1H), 8.45 (t, *J* = 5.1 Hz, 1H), 8.20 – 7.99 (m, 3H), 7.78 (dd, *J* = 6.8, 2.3 Hz, 1H), 7.74 – 7.67 (m, 1H), 7.60 – 7.52 (m, 1H), 7.49 – 7.38 (m, 2H), 7.37 – 7.29 (m, 2H), 3.84 – 3.71 (m, 1H), 3.46 – 3.30 (m, 3H), 3.30 – 3.14 (m, 1H), 1.34 (d, *J* = 7.0 Hz, 3H). ES$^+$ calc. for $C_{18}H_{20}F_2N_3O_2$ [M+H]$^+$: 348.2. Found 348.2.

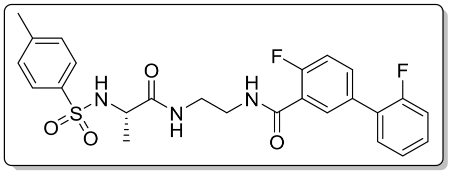

PKS21290: The title compound was synthesized by following the general procedure for *N*-sulfonamide preparation of **PKS21285** (16.0 mg, 35 μmol) with 4-methylbenzenesulfonyl chloride (9.9 mg, 52 μmol). After completion of reaction, dichloromethane was evaporated and crude was purified by preparative LCMS to give product (12.4 mg, 71%) as white solid. ^{1}H NMR (500 MHz, DMSO-d_6) δ 8.36 – 8.30 (m, 1H), 7.99 (t, *J* = 5.7 Hz, 1H), 7.89 (br, 1H), 7.78 – 7.75 (m, 1H), 7.72 – 7.68 (m, 1H), 7.65 (d, *J* = 8.3 Hz, 2H), 7.56 (td, *J* = 7.9, 1.9 Hz, 1H), 7.48 – 7.38 (m, 2H), 7.37 – 7.29 (m, 4H), 3.70 – 3.60 (m, 1H), 3.27 – 3.01 (m, 4H), 2.36 (s, 3H), 1.03 (d, *J* = 7.1 Hz, 3H). ^{13}C NMR (126 MHz, DMSO-d_6) δ 171.5, 163.5, 159.0 (d, *J* = 245.7 Hz), 158.8 (d, *J* = 252.8 Hz), 142.6, 138.1, 132.7 (d, *J* = 7.8 Hz), 131.3, 130.8, 130.4, 130.0 (d, *J* = 8.7 Hz), 129.4, 126.6, 126.6 (d, *J* = 12.1 Hz), 125.1 (d, *J* = 2.9 Hz), 124.2 (d, *J* = 14.6 Hz), 116.6 (d, *J* = 22.0 Hz), 116.2 (d, *J* = 23.5 Hz),

52.0, 38.9, 38.1, 21.0, 18.8. ^{19}F NMR (471 MHz, DMSO-d_6) δ -117.8 – -117.9 (m), -120.8 – -120.9 (m). HRMS calc. for $C_{25}H_{26}F_2N_3O_4S$ $[M+23]^+$: 524.1432. Found: 524.1451.

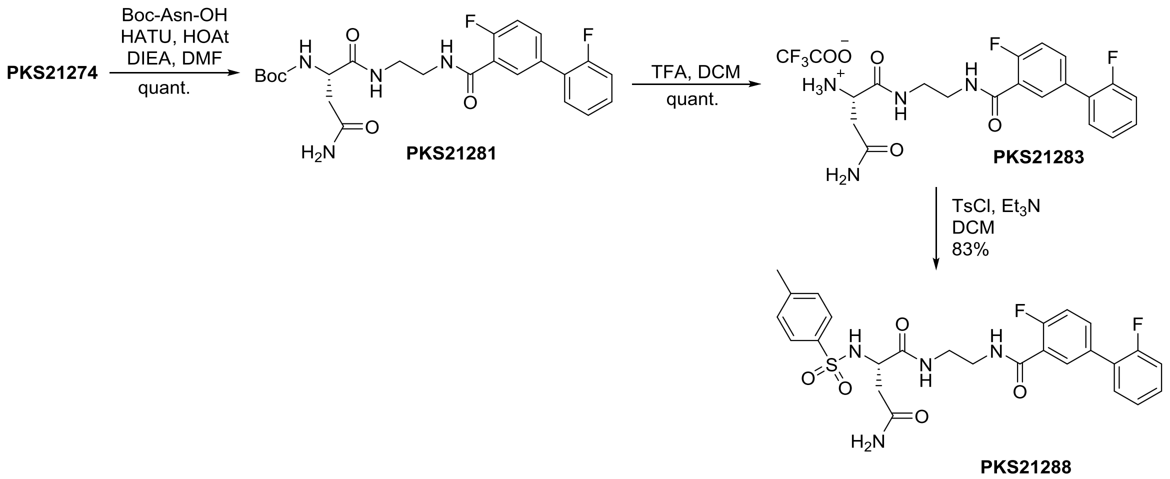

Scheme 9

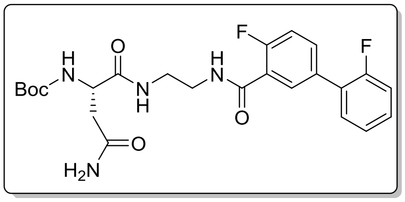

PKS21281: The title compound was synthesized by following the general procedure for HATU mediated coupling of Boc-Asn-OH (23.2 mg, 100 μmol) and **PKS21274** (39.0 mg, 100 μmol). After completion of reaction, mixture was purified by preparative LCMS to give product (43.6 mg, 89%) as white solid. ^{1}H NMR (500 MHz, DMSO-*d6*) δ 8.36 (t, *J* = 5.6 Hz, 1H), 7.95 (t, *J* = 5.6 Hz, 1H), 7.79 (dd, *J* = 7.1, 2.5 Hz, 1H), 7.72 – 7.67 (m, 1H), 7.57 (td, *J* = 7.9, 1.7 Hz, 1H), 7.49 – 7.41 (m, 1H), 7.40 (dd, *J* = 10.3, 8.6 Hz, 1H), 7.38 – 7.28 (m, 2H), 7.28 – 7.21 (m, 1H), 6.87 (s, 1H), 6.81 (d, *J* = 8.0 Hz, 1H), 4.21 – 4.14 (m, 1H), 3.37 – 3.22 (m, 3H), 3.21 – 3.14 (m, 1H), 2.43 (dd, *J* = 15.0, 5.3 Hz, 1H), 2.35 (dd, *J* = 15.0, 8.1 Hz, 1H), 1.34 (s, 9H). ES^+ calc. for $C_{24}H_{29}F_2N_4O_5$ $[M+23]^+$: 513.1918. Found 513.1925.

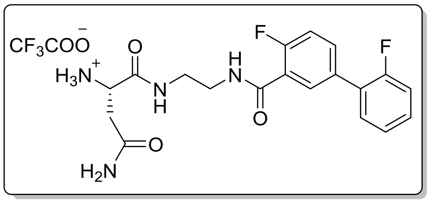

PKS21283: The title compound was synthesized by following the general procedure for Boc-deprotection of **PKS21281** (30.0 mg, 61 μmol). Isolated crude was dried under vacuum and triturated with diethyl ether to give a white solid. Diethyl ether was decanted and white solid was dried under vacuum to give product (30.8 mg, quant.). Product was used in next step

without further purification. ^{1}H NMR (500 MHz, DMSO-*d6*) δ 8.54 (t, *J* = 5.3 Hz, 1H), 8.49 – 8.41 (m, 1H), 8.11 (d, *J* = 5.2 Hz, 3H), 7.84 – 7.76 (m, 1H), 7.74 – 7.69 (m, 1H), 7.65 (br, 1H), 7.57 (td, *J* = 7.9, 1.7 Hz, 1H), 7.48 – 7.38 (m, 2H), 7.37 – 7.28 (m, 2H), 7.23 (br, 1H), 4.05 – 3.95 (m, 1H), 3.43 – 3.29 (m, 3H), 3.29 – 3.20 (m, 1H), 2.70 (dd, *J* = 16.8, 4.6 Hz, 1H), 2.58 (dd, *J* = 16.8, 8.3 Hz, 1H). ES^{+} calc. for $C_{19}H_{21}F_2N_4O_3$ $[M+H]^+$: 391.2. Found 391.3.

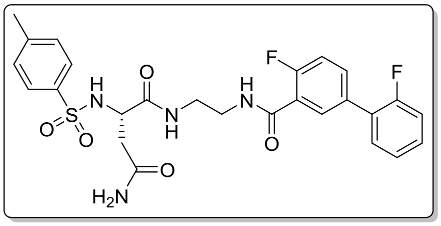

PKS21288: The title compound was synthesized by following the general procedure for sulfonamide preparation of **PKS21283** (15.1 mg, 30 µmol) with 4-methylbenzenesulfonyl chloride (11.4 mg, 60 µmol). After completion of reaction, dichloromethane was evaporated and crude was purified by preperative LCMS to give product (13.6 mg, 83%) as white solid. ^{1}H NMR (500 MHz, DMSO-d_6) δ 8.33 – 8.24 (m, 1H), 7.97 (t, *J* = 5.8 Hz, 1H), 7.85 (br, 1H), 7.78 (dd, *J* = 7.1, 2.4 Hz, 1H), 7.74 – 7.68 (m, 1H), 7.64 (d, *J* = 8.3 Hz, 2H), 7.57 (td, *J* = 7.9, 1.9 Hz, 1H), 7.48 – 7.38 (m, 2H), 7.36 – 7.28 (m, 4H), 7.27 (d, *J* = 2.3 Hz, 1H), 6.84 (d, *J* = 2.3 Hz, 1H), 4.04 – 3.95 (m, 1H), 3.25 – 3.09 (m, 2H), 3.09 – 2.92 (m, 2H), 2.39 – 2.30 (m, 1H), 2.35 (s, 3H), 2.21 (dd, *J* = 15.1, 6.8 Hz, 1H); ^{13}C NMR (126 MHz, DMSO-d_6) δ 170.7, 170.1, 163.4, 159.0 (d, *J* = 246.9 Hz), 158.8 (d, *J* = 251.2 Hz), 142.5, 138.1, 132.7 (d, *J* = 8.3 Hz), 131.3, 130.8, 130.3, 130.0 (d, *J* = 7.8 Hz), 129.2, 126.7, 126.6, 125.3 – 124.9 (m), 124.2 (d, *J* = 14.1 Hz), 116.6 (d, *J* = 23.4 Hz), 116.2 (d, *J* = 22.1 Hz), 53.4, 38.8, 38.2, 38.2, 21.0; ^{19}F NMR (471 MHz, DMSO-d_6) δ -117.7 – -117.9 (m), -120.8 – -120.9 (m). HRMS calc. for $C_{26}H_{27}F_2N_4O_5S$ $[M+23]^+$: 567.1490. Found: 567.1490.
